# Anti-CD3 mAb treatment reshapes infiltrating T and **β** cells in the islets in autoimmune diabetes

**DOI:** 10.1172/jci.insight.192755

**Published:** 2026-01-23

**Authors:** Ying Wu, Maxwell Spurrell, Ana Lledó-Delgado, Songyan Deng, Dejiang Wang, Yang Liu, Mahsa Nouri Barkestani, Ana Luisa Perdigoto, Kevan C. Herold

**Affiliations:** 1 Department of Immunobiology,; 2 Department of Pathology,; 3 Cancer Center,; 4 Department of Neurology, and; 5 Department of Internal Medicine, Yale School of Medicine, Yale University, New Haven, Connecticut, USA.

**Keywords:** Endocrinology, Immunology, Diabetes, T cells, Tolerance

## Abstract

Treatment with anti-CD3 monoclonal antibody (mAb) can delay or prevent type 1 diabetes in mice and humans by modulating the immune-mediated destruction of β cells. A single course of treatment may have lasting efficacy, but the mechanisms that account for these prolonged effects, i.e., “operational tolerance,” are not clear. Here, we used paired single-cell RNA and T cell receptor sequencing to characterize islet-infiltrating T cells and their counterpart in paired pancreatic lymph nodes from anti-CD3 mAb–treated nonobese diabetic (NOD) mice in remission. We found that after anti-CD3 mAb treatment, T cells that infiltrate the islets are more heterogeneous and have hybrid features including characteristics of T stem cell–like memory and reduced effector function compared with those from untreated prediabetic NOD mice. Autoantigen-reactive CD8^+^ T cells persist after treatment, but they also show features of stemness and reduced pathogenicity. Our findings describe the reshaping of islet-infiltrating and autoreactive T cells and β cells that lead to operational, but tenuous, tolerance to autoimmune diabetes following anti-CD3 mAb treatment.

## Abstract 2

Anti-CD3 mAb delays or prevents type 1 diabetes (T1D) by modulating the immune mediated destruction of beta cells. Our findings described the reshaping of islet-infiltrating T cells and beta cells that lead to operational, but tenuous tolerance to autoimmune diabetes following anti-CD3 mAb treatment.

## Introduction

Type 1 diabetes (T1D) is an autoimmune disease in which T cells progressively destroy insulin-producing β cells in the islets of Langerhans. In humans at risk for, or with, T1D, the humanized FcR non-binding anti-CD3 monoclonal antibody (mAb) teplizumab attenuates immune-mediated destruction of β cells. Clinical studies showed that a single or two 14-day courses of teplizumab preserved function of β cells and significantly increased C-peptide levels (1, 2). The anti-CD3 mAb does not bind the FcR but delivers a partial agonist signal (3).

A course of drug treatment has effects that are sustained beyond the 12 or 14 days that the drug is administered or can be found on the surfaces of circulating T cells. For example, in patients with preclinical, stage 2 T1D, the delay in the median time to diagnosis with clinical stage 3 T1D was 24.9 months after the 2-week study drug treatment: from 27.3 months with placebo to 52.2 months with teplizumab (4). The mechanisms of treatment that establish operational tolerance and these prolonged clinical effects are not clear.

Previous studies in patients suggested that teplizumab induces, in CD8^+^ T cells, a partial exhaustion phenotype, with a peak level at about 6 months of teplizumab treatment (5, 6). These partially exhausted T cells have reduced production of TNF and IFN-γ when they are activated ex vivo, and are characterized by coexpression of EOMES, KLRG1, and TIGIT. However, this phenotype does not persist, and the frequency of these cells declines to baseline about 1–1.5 years after a single course of the drug treatment (6). The decline in these cells suggests that these findings alone may not explain the extended efficacy that is seen in some patients (4). Moreover, the effects of the drug treatment in peripheral blood may not reflect what happens at the site of disease activity — in the islets or draining lymph nodes.

In addition, not all patients who are treated with teplizumab respond to the treatment, and the duration of efficacy wanes in some. In the teplizumab prevention trial, while the median time to diagnosis with clinical disease was 52.2 months, some individuals did not develop clinical disease for more than 120 months (10 years), whereas others progressed in 6.5 months after treatment (4). The basis for the long-term responses in some and failure in others is not clear.

The nonobese diabetic (NOD) mouse is a model of spontaneous T1D and has been used extensively to analyze the mechanisms of human disease and treatments. Chatenoud et al. showed that anti-CD3 mAb can induce long-term tolerance in NOD mice treated at the time of hyperglycemia. They observed that T cells disappeared from the islets within days after the mAb treatment but reappeared in the islets after about 2 weeks (7, 8). Because hyperglycemia did not recur in the treated mice, this observation suggested that the islet-infiltrating cells had been rendered non-pathogenic and tolerant to the islet cells. In this study, we characterized the molecular phenotype and repertoire of these T cells that infiltrate islets after anti-CD3 mAb treatment in NOD mice with established remission as well as the effects on β cells after the treatment. We find reshaping of the islet-infiltrating repertoire after anti-CD3 mAb but also changes in autoantigen-reactive T cells that suggest reduced cytolytic function and exhaustion. These investigations suggest mechanisms whereby treatment with a partial agonist mAb against CD3 may induce long tolerance.

## Results

### Insulitis in islets from remitter NOD mice after anti-CD3 treatment.

**Figure 1:**
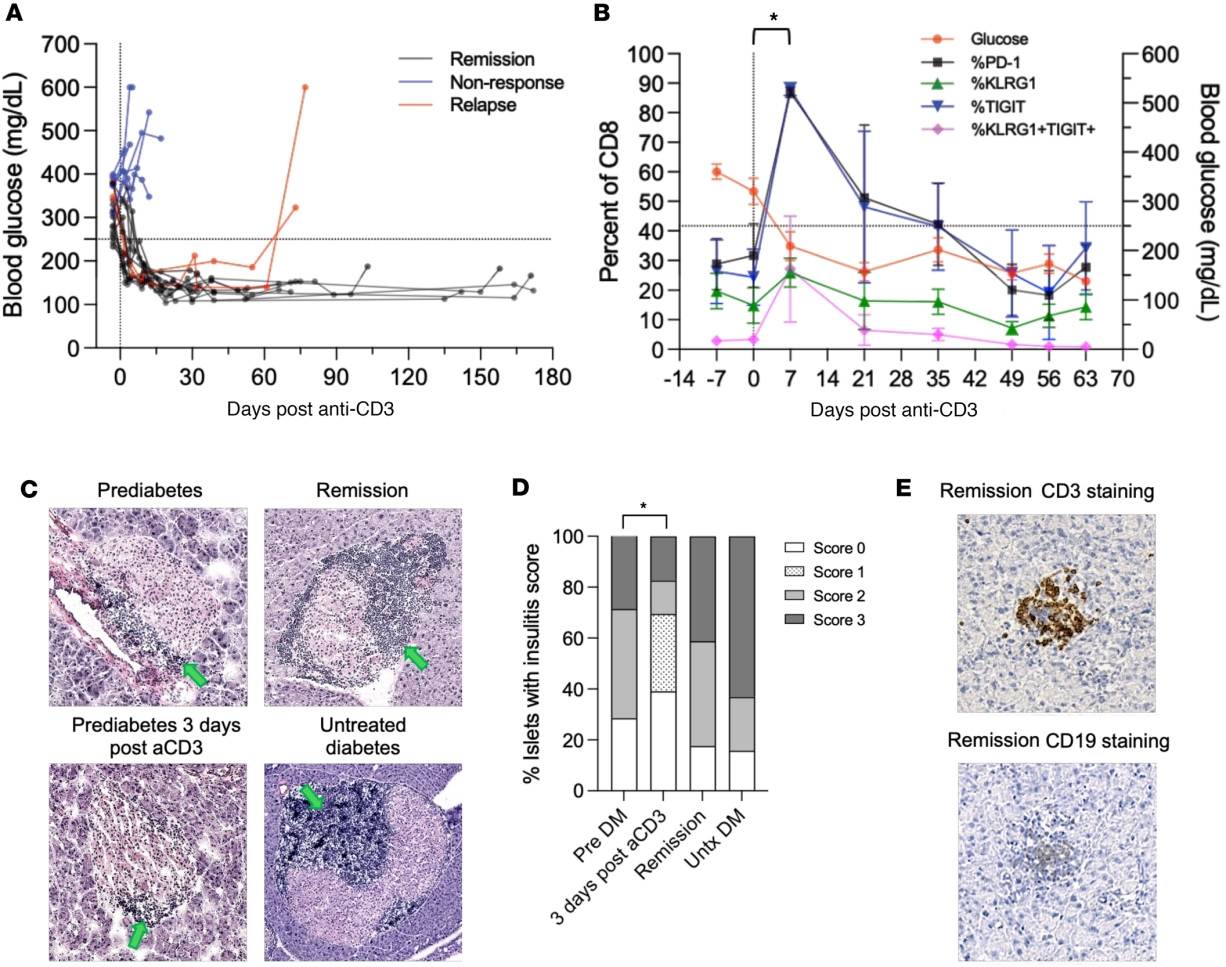
Peripheral blood and islet-infiltrating cells in remitter NOD mice after anti-CD3 mAb treatment. (**A**) Newly diabetic NOD mice (*n* = 24) were treated with the F(ab′)_2_ fragments of anti-CD3 mAb 145-2C11 (5 μg/d for 3 or 4 days, i.p.) and were considered in remission with blood glucose lower than 250 mg/dL for ≥9 weeks after the treatment (remission: *n* = 14; non-response: *n* = 8; relapse: *n* = 2). Glucose levels after anti-CD3 mAb treatment are shown. (**B**) CD8^+^ T cells from blood of NOD mice with remission after anti-CD3 treatment were examined for expression of PD-1, KLRG1, and TIGIT by flow cytometry (dashed line = 250 mg/dL for blood glucose level) (*P = 0.0133 for PD-1 expression and *P = 0.0294 for TIGIT expression at day 7 vs. day 0, ANOVA for repeated measures with Dunnett’s multiple-comparison test). (**C**) Representative H&E images show insulitis in islets from an untreated prediabetic NOD mouse (insulitis score = 2), a remitter NOD mouse after anti-CD3 treatment (insulitis score = 2), a prediabetic NOD mouse 3 days after anti-CD3 treatment (insulitis score = 1), and an untreated diabetic NOD mouse (insulitis score = 3) (green arrows point at infiltrates). Original magnification, ×10. (**D**) Bar chart showing insulitis scores of untreated prediabetic (Pre DM; DM, diabetes mellitus) NOD mice (14 islets from 3 mice), prediabetic NOD mice 3 days after anti-CD3 treatment (46 islets from 3 mice), anti-CD3–treated remitter NOD mice (17 islets from 4 mice), and untreated diabetic (Untx DM) NOD mice (19 islets from 5 mice). A significant difference was found comparing all the 4 conditions (χ^2^ test, *P* < 0.0001). Insulitis was significantly lower 3 days after treatment in anti-CD3–treated prediabetic versus untreated prediabetic NOD mice (χ^2^ test, *P = 0.018). (**E**) Immunohistochemical images showing islets from a remitter NOD mouse stained by anti-CD3 (in brown) and anti-CD19, respectively. Original magnification, ×10.

We treated NOD mice with newly confirmed hyperglycemia with FcR non-binding anti-CD3 mAb at a lower dose than has conventionally been used to evaluate the effects of antibody treatment, with the goal of modeling treatment of patients. At this dosing (5 μg/d for 3 or 4 days), hyperglycemia was reversed and persisted in about 60% of mice by day 30 (Figure 1A).

This treatment resulted in changes of CD8^+^ T cells in the peripheral blood that were similar to those in patients. We found increased expression of KLRG1 and TIGIT on CD8^+^ T cells in the peripheral blood, and, as had been seen in some patients, there was an abrupt increase of PD-1 expression acutely after treatment. These changes in phenotype declined to pretreatment levels by day 50 (Figure 1B) (5, 6).

We examined the histology of the islets after reversal of hyperglycemia. Consistent with studies by Chatenoud et al. (7, 8), we observed significantly reduced insulitis in the islets from NOD mice 3 days after anti-CD3 treatment when compared with untreated prediabetic mice (*P* = 0.018) (Figure 1, C and D). At later times, with anti-CD3 mAb–induced remission, we found infiltration of T cells into the islets of NOD mice (Figure 1, C and E). We identified islets with focal and peripheral infiltration but did not find a difference in the severity of insulitis in the mice with remission after anti-CD3 mAb compared with untreated prediabetic mice (Figure 1D).

**Figure 2:**
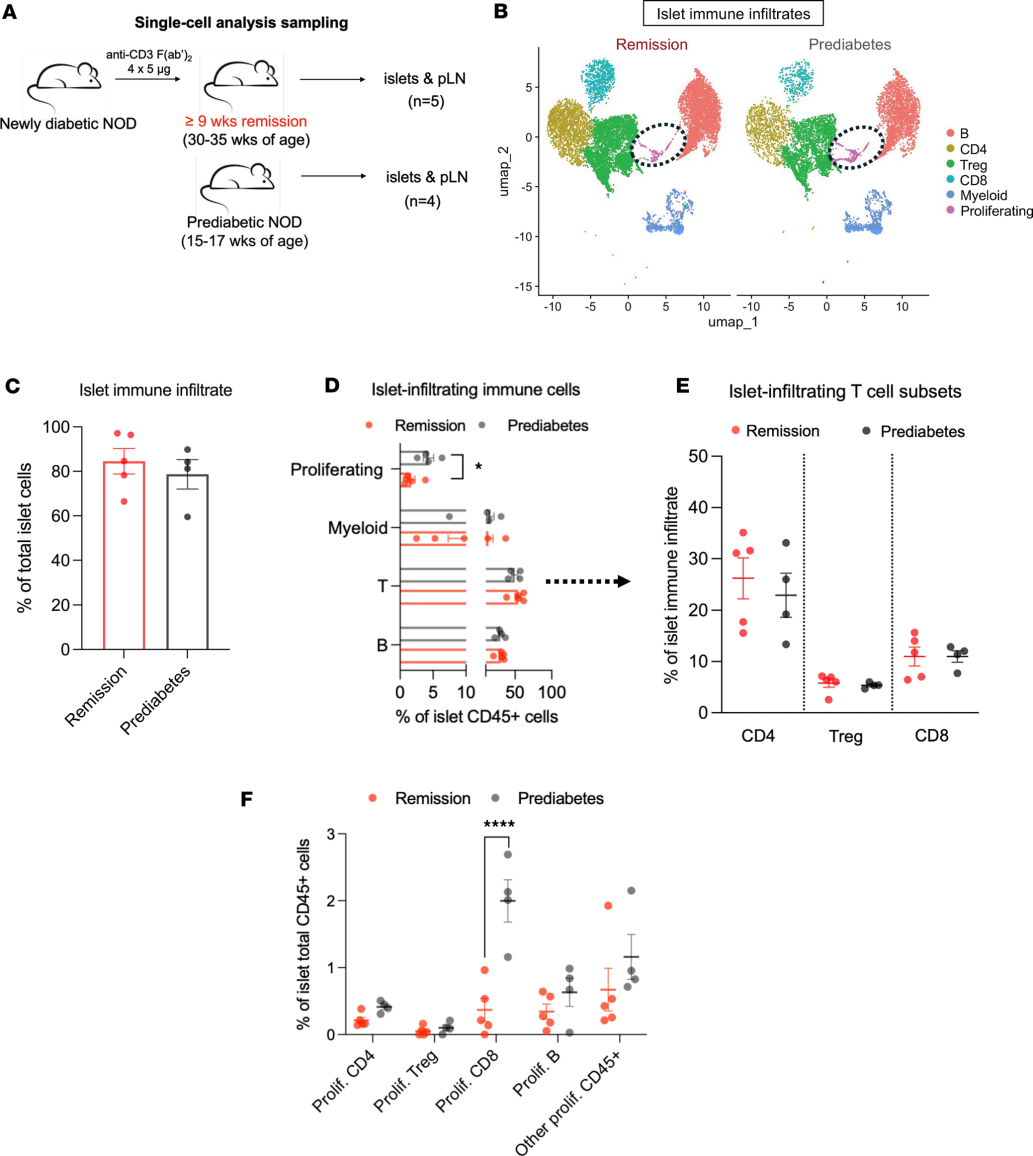
Single-cell RNA-Seq analysis of islets from anti-CD3–treated remitter NOD mice. (**A**) Islets and ppLNs freshly isolated from anti-CD3–treated remitter (*n* = 5) or prediabetic NOD mice (*n* = 4) were processed and analyzed by scRNA-Seq. (**B**) UMAP visualization of immune cell clusters in the islets and ppLNs, integrated from all the mice in single-cell analysis. The ovals identify proliferating cells that are identified in **F**. (**C**) Percentage of all the immune cells infiltrating islets of remitter or prediabetic NOD mice. Immune cells were identified based on expression of *Ptprc* (encoding CD45). Each symbol represents 1 individual mouse. No significant difference was found between the 2 conditions. (**D**) Percentage of each immune cell cluster (proliferating, myeloid, T or B cells; T cells were identified based on expression of *Cd3e/g/d*) among total islet infiltrates in remitter or prediabetic NOD (proliferating cluster: *P = 0.026; all other clusters: NS, unpaired, 2-tailed *t* test). (**E**) Islet-infiltrating T cells were subgrouped into CD4^+^, Treg, and CD8^+^ cells. No significant difference was found in percentage of each T subset among islet immune infiltrates between the 2 conditions. (**F**) Scatterplot showing percentage of each proliferating subset among total CD45^+^ immune cells in the islets (****P < 0.0001, 2-way ANOVA). All data are shown as mean ± SEM.

Although immune cells reappeared in the islets, diabetes did not recur after treatment. To identify the composition of these non-pathogenic islet-infiltrating cells, we performed single-cell RNA-Seq (scRNA-Seq) analysis of the islets from NOD mice in remission and compared them with those in prediabetic mice that were not treated with anti-CD3 mAb. We also studied paired pancreatic lymph nodes (ppLNs) from the same mice (experimental design shown in Figure 2A).

Major clusters of immune cells were visualized using uniform manifold approximation and projection (UMAP) and annotated based on canonical markers (Figure 2B and Supplemental Figure 1A; supplemental material available online with this article; https://doi.org/10.1172/jci.insight.192755DS1). The scRNA-Seq analysis was performed using a total of 11,645 and 8,200 islet-infiltrating CD45^+^ immune cells from remitter and prediabetic control mice, respectively, and a total of 15,044 and 9,199 immune cells from ppLNs of the same mice. The frequencies of the major immune cell populations in the islets from anti-CD3–treated NOD remitters were similar to those in the ppLNs, consisting of T cells, B cells, myeloid cells, Tregs, and a cluster of mixed immune cells showing proliferating features (i.e., *Stmn1*, *Birc5*, *Lmnb1*, *Ki67*, *Nuf2*, or *Kif4*; Figure 2A and Supplemental Figure 1A). B cells and myeloid cells were clustered as a single population and defined by canonical markers (Figure 2B and Supplemental Figure 1A). In accord with the previously reported histologic data, by transcriptome analysis, the islet infiltrates of remitted NOD mice were dominated by CD4^+^ and CD8^+^ T cells, but there were no significant differences in their frequency in the islets from remitters and prediabetic mice (Figure 2, C–E) (7, 8). However, there was a significantly reduced frequency of proliferating cells in the islets from mice with remission (*P* < 0.05) (Figure 2D). This proliferative cluster was a mixed population, predominantly composed of T cells with a small part of B and myeloid cells. The majority of the proliferating cells in the islets of prediabetic NOD mice were CD8^+^ T cells, and this population was significantly reduced in frequency in the islets of remitter NOD mice (Figure 2F). Interestingly, these findings were restricted to the islets, as we did not find these same differences in the frequency of T and B cells or in proliferating cells in the ppLNs (Supplemental Figure 1C).

### Transcriptional features and repertoire of islet-infiltrating CD4^+^ T cells.

**Figure 3:**
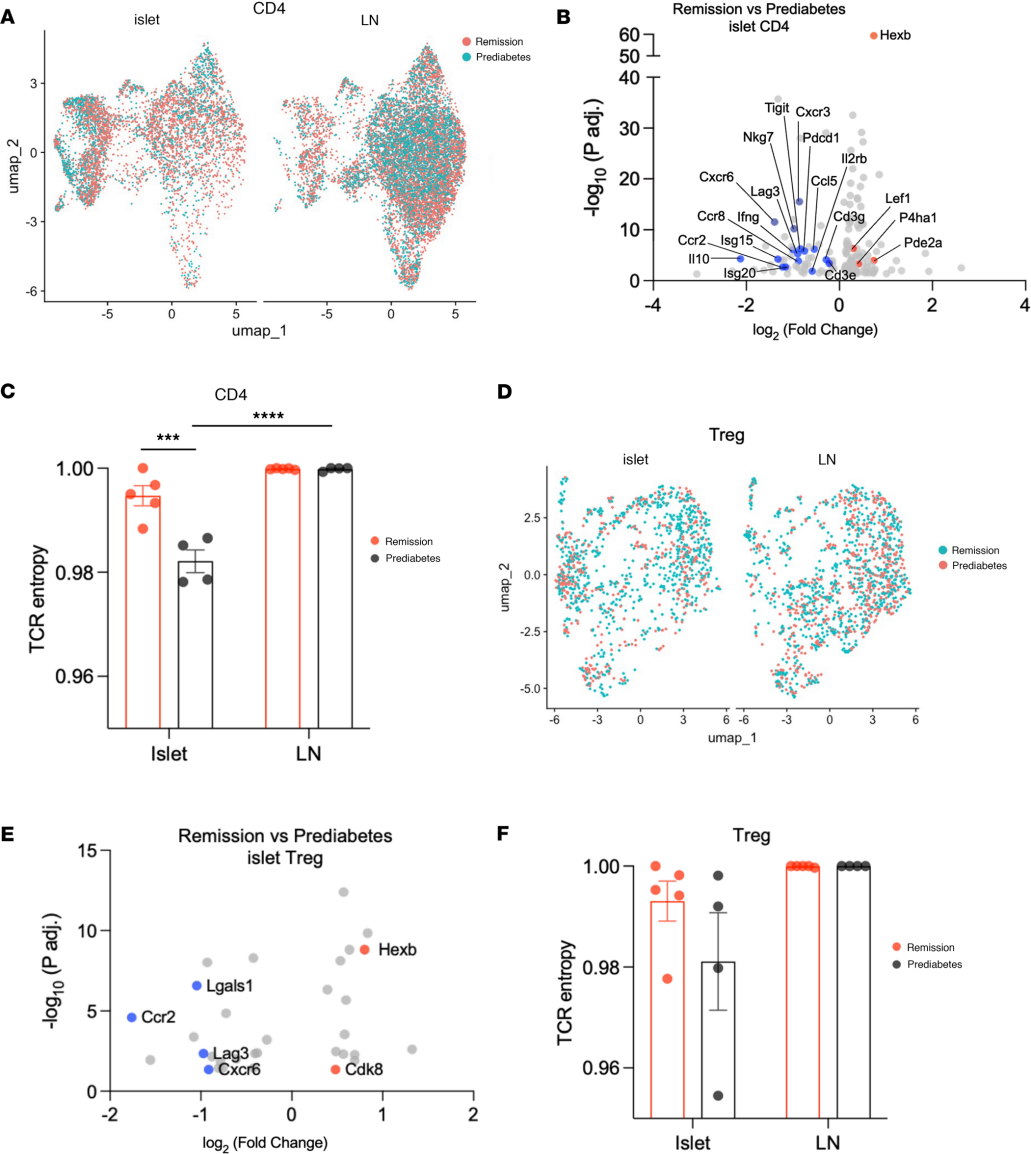
Transcriptional features of islet-infiltrating CD4^+^ T cells. (**A**) UMAP showing CD4^+^ T cells in the islets or ppLNs of anti-CD3–treated remitter and prediabetic NOD mice, colored by conditions. (**B**) Volcano plot showing differentially expressed genes (DEGs) of islet-infiltrating CD4^+^ T cells between remitter and prediabetic NOD (adjusted *P* values < 0.05). Upregulated genes of note in remitters are highlighted in red and downregulated genes in blue. (**C**) Repertoire diversity of CD4^+^ T cells measured by Shannon entropy index (entropy index scaled to 1; mean ± SEM). Each symbol represents 1 individual mouse (***P < 0.001, ****P < 0.0001, 2-way ANOVA with Tukey’s multiple-comparison test). (**D**) UMAP showing Tregs in the islets or ppLNs of anti-CD3–treated remitter and prediabetic NOD mice, colored by conditions. (**E**) Volcano plot showing DEGs of islet-infiltrating Tregs between remitter and prediabetic NOD (adjusted *P* values < 0.05). (**F**) Repertoire diversity of Tregs measured by Shannon entropy index (entropy index scaled to 1). Each symbol represents 1 individual mouse (mean ± SEM).

We compared gene expression in the islet-infiltrating CD4^+^ T cells from the treated remitter and prediabetic NOD mice (Figure 3, A and B). There were 177 differentially expressed genes (DEGs) identified, which included significantly reduced (i.e., adjusted *P* value < 0.05) expression of genes of the CD3 complex (*Cd3e/g*); chemokines, cytokines, and ligands (*Ccl5*, *Cxcr3*, *Ccr8*, *Ccr2*, *Cxcr6*); and IFN-γ and type II interferon genes (*Ifng*, *Isg15*, *Isg20*) (Figure 3B and Supplemental Table 1). Curiously, genes associated with immune regulation (*Pdcd1*, *Tigit*, *Lag3*, *Il10*) were also significantly reduced in remitter NOD compared with prediabetic control mice (Figure 3B). Ingenuity Pathway Analysis (IPA) of the DEGs showed that only the integrin-linked kinase (ILK) pathway, which is associated with cell migration, was increased in the remitter mice (*z* score = 2.24, *P* = 0.0002).

In the ppLNs from the same mice, we found 367 DEGs in the CD4^+^ T cells between remitter and prediabetic NOD mice (Supplemental Table 2). The expression of some genes associated with CD4^+^ T cell activation and survival (*Bmpr1a*, *Gpr68*) was significantly increased, whereas others (*Tcf7*, *Il7r*, *Sell*) were reduced (Supplemental Figure 3B). Likewise, an IPA analysis showed that pathways associated with CD4^+^ T cell activation and effector function were significantly increased in the ppLNs (i.e., *z* score > 2, *P* < 0.05), whereas, surprisingly in remitters, PPAR, PTEN, and PD-1/PD-L1 signaling, associated with T cell regulation, was significantly reduced (i.e., *z* score <–2, *P* < 0.05) (Supplemental Figure 3C).

We compared the repertoire of the islet-infiltrating CD4^+^ T cells in the remitters and prediabetic mice to that of the ppLNs using 5′ sequencing. There was significantly decreased entropy in the islet-infiltrating CD4^+^ T cells of the prediabetic mice versus ppLNs (*P* < 0.0001), consistent with clonal expansion of the cells in the islets. In addition, we found significantly increased entropy among the islet-infiltrating CD4^+^ T cells in remitters when comparing the islets of remitters versus prediabetic controls (*P* < 0.001) (Figure 3C).

The frequency of islet-infiltrating Tregs (identified by expression of *Cd4*, *Foxp3*, *Il2ra*, *Ctla4*, *Ikzf2*, and *Lag3*; Supplemental Figure 1A) was similar in the remitters and the prediabetic mice, but we found 32 DEGs in the islet-infiltrating Tregs (Figure 3, D and E, and Supplemental Table 3). These differences included increased expression of *Hexb* (for CD4^+^ T cell activation) (9) and *Cdk8* (an inhibitor of Treg differentiation) (10), whereas other genes associated with Tregs (*Lag3*, *Lgals1*) and chemokine receptors (*Cxcr6* and *Ccr2*) showed significantly reduced expression (Figure 3E). Repertoire analysis also showed a numerically greater T cell receptor (TCR) diversity among this subset of CD4^+^ T cells in the islet-infiltrating Tregs from remitters, but this difference was not statistically significant (Figure 3F). In the ppLNs from the same mice, we found similar frequencies of Tregs and 8 DEGs between remitters and prediabetic mice (Supplemental Table 4). We did not identify any pathways with significant differences by IPA analysis.

### Transcriptional features and repertoire of islet-infiltrating CD8^+^ T cells.

**Figure 4:**
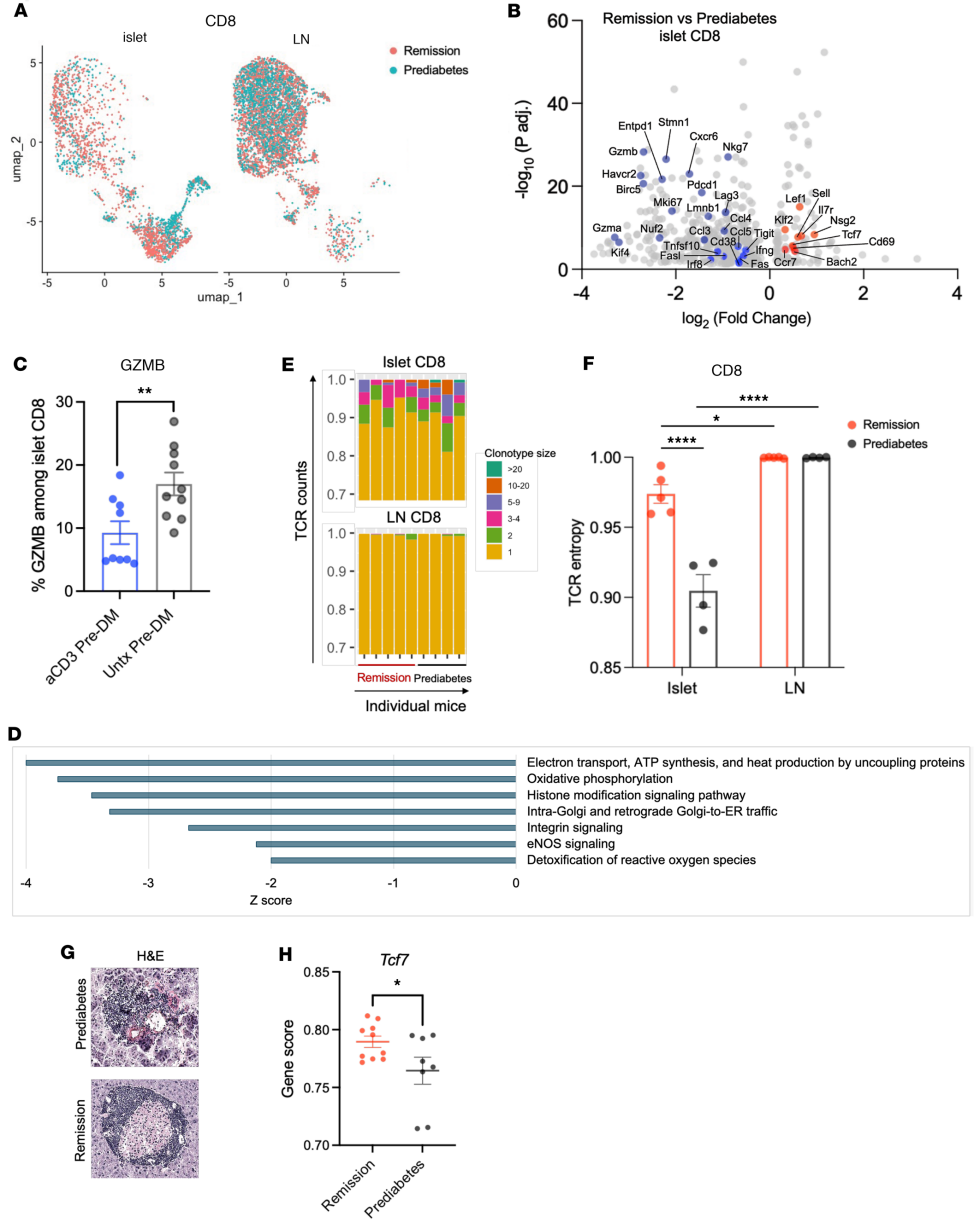
Changes in intra-islet CD8^+^ T cells after anti-CD3 mAb treatment. (**A**) UMAP showing CD8^+^ T cells in islets or ppLNs of anti-CD3–treated remitter and prediabetic NOD mice, colored by conditions. (**B**) Volcano plot showing DEGs of islet-infiltrating CD8^+^ T cells between remitter and prediabetic NOD (adjusted *P* values < 0.05). Upregulated genes of note in remitters are highlighted in red and downregulated genes in blue. (**C**) Bar chart showing percentage of granzyme B^+^ islet-infiltrating CD8^+^ T cells of anti-CD3–treated versus untreated prediabetic NOD mice by flow cytometry. Each symbol represents an individual mouse (**P = 0.008, unpaired, 2-tailed *t* test). (**D**) Ingenuity Pathway Analysis of islet-infiltrating CD8^+^ T cells in remitter versus prediabetic NOD mice (*z* scores ≤ –2, *P* values < 0.05). (**E**) Bar graphs showing TCR clonotype frequencies of CD8^+^ T cells. Raw count of each clonotype was divided by total count, and result is shown with normalization by scaling to 1. Each bar represents 1 individual mouse. Colors denote clonotype size based on raw count. (**F**) Repertoire diversity of CD8^+^ T cells was measured by Shannon entropy index (entropy index scaled to 1; data shown as mean ± SEM). Each symbol represents 1 individual mouse (*P = 0.038, ****P < 0.0001, 2-way ANOVA with Tukey’s multiple-comparison test). (**G** and **H**) Spatial-ATAC-seq of islets from remitter and prediabetic mice. (**G**) H&E images feature 1 representative islet of either condition with similar degree of insulitis (original magnification, ×10). (**H**) Summary bar charts showing gene scores for *Tcf7* expression (a total of 10 islets from 3 remitter NOD mice and a total of 8 islets from 3 prediabetic NOD mice evaluated by 3 repeated measurements with mixtures of islets from both groups; *P = 0.034, Mann-Whitney test). Data are shown as mean ± SEM.

We identified a greater number of DEGs (i.e., 714) among the CD8^+^ T cells that infiltrate the islets from remitters versus prediabetic NOD mice (Figure 4, A and B, and Supplemental Table 5). In the remitters, there was significantly reduced expression of genes in cell proliferation pathways such as *Mki67*, *Nuf2*, *Kif4*, *Stmn1*, *Birc5*, *Nkg7*, and *Lmnb1*. There was significantly reduced expression of genes associated with terminally differentiated effector CD8^+^ cells such as *Ifng*, *Gzma*, *Fasl*, and *Tnfsf10* as well as *Gzmb*. We confirmed the latter finding by flow cytometry indicating that these cells had reduced cytolytic activity, by comparing anti-CD3–treated prediabetic versus untreated prediabetic NOD mice (Figure 4C) (11).

Interestingly, multiple genes that identify T cell stemness, such as *Lef1*, *Ccr7*, *Sell* (*Cd62l*), *Bach2*, *Il7r*, *Tcf7*, *Klf2*, *Cd69*, and *Nsg2*, were significantly increased, and the expression of *Irf8* (12), which drives the effector differentiation of CD8^+^ T cells, was significantly reduced (Figure 4B). Surprisingly, some genes whose expression was associated with CD8^+^ T cell exhaustion or non-responsiveness in CD8^+^ cells in the peripheral blood, such as *Pdcd1*, *Tigit*, and *Klrg1* (Figure 1B), as well as *Lag3*, *Cd38*, and *Havcr2* (encoding Tim3), were significantly reduced in the islet-infiltrating cells (Figure 4B).

An IPA analysis showed that pathways related to cellular metabolism and expansion, including oxidative phosphorylation, mitochondrial activity, and electron transport pathways, were significantly reduced in the remitters (Figure 4D). Integrin signaling was also significantly decreased, consistent with reduced expression of *Ccl3*, *Ccl4*, *Ccl5*, and *Cxcr6* (Figure 4, B and D). In the ppLNs, we identified 119 DEGs in CD8^+^ T cells from remitter versus prediabetic mice, but we did not find any shared pathways between the islets and lymph nodes (Supplemental Table 6).

Like the CD4^+^ cells, the repertoire of the islet-infiltrating CD8^+^ T cells showed significantly reduced entropy in comparison with ppLNs of the prediabetic mice (*P* < 0.0001). There was significantly greater entropy in the anti-CD3–treated remitter versus prediabetic mice in the islets (*P* < 0.0001). The counterparts in the ppLNs were all single clones (*P* < 0.001) (Figure 4, E and F). Therefore, these data indicate that, overall, the expansion of selected CD4^+^ or CD8^+^ T cells that was seen in the prediabetic mice was reduced in the CD8^+^ T cells after anti-CD3 mAb treatment.

TCF1 (encoded by *Tcf7*), a transcription factor whose expression is epigenetically regulated by Notch binding and is associated with self-renewal and stemness of T cells, was increased (13, 14). To confirm the expression level of *Tcf7* between the 2 conditions in situ, we performed spatial assay for transposase-accessible chromatin using sequencing (ATAC-seq) (spatial-ATAC-seq) of islets from the remitters and prediabetic mice with similar degree of insulitis. We generated a computational metric, “gene score,” that estimates a gene’s activity or expression level based on the chromatin accessibility of its regulatory regions. We found a significantly higher gene score of *Tcf7* in the islets from the remitters versus prediabetic mice (*P* = 0.034) (Figure 4, G and H).

### Autoantigen-reactive CD8^+^ T cells in the islets.

**Figure 5:**
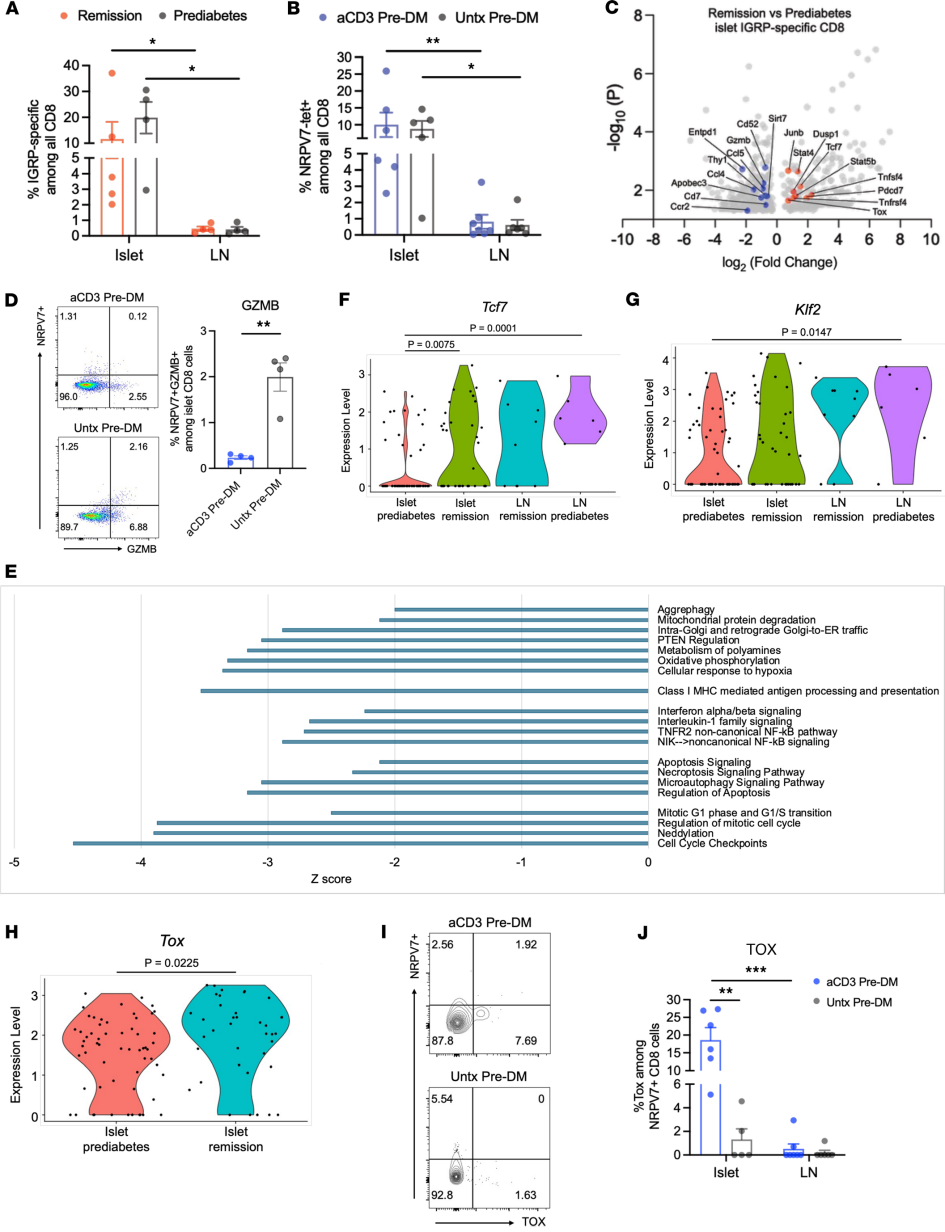
Autoantigen-reactive CD8^+^ T cells in islets show transcriptional features of exhaustion and stemness. (**A**) Percentage of NRPV7^+^ T cells among CD8^+^ T cells in islets or ppLNs of anti-CD3–treated remitter or prediabetic NOD mice by scRNA-seq analysis. Each symbol represents 1 mouse. Comparison between sites was performed by Mann-Whitney test (remission, *P = 0.016; prediabetes, *P = 0.029). (**B**) Percentage of NRPV7^+^ cells among CD8^+^ T cells by site by flow cytometry. Prediabetic NOD mice were treated with F(ab′)2 fragments of anti-CD3 mAb 145-2C11. Two weeks after the treatment, islets and ppLNs were isolated for flow cytometry in comparison with counterparts from age-matched untreated prediabetic NOD mice. Each symbol represents 1 mouse (*P < 0.05, **P < 0.01). (**C**) Volcano plot showing DEGs of islet-infiltrating NRPV7^+^ CD8^+^ T cells between remitter and prediabetic NOD (*P* < 0.05). Upregulated genes are highlighted in red and downregulated genes in blue. (**D**) Staining for and percentage of granzyme B^+^ NRPV7^+^ cells among CD8^+^ T cells determined by flow cytometry using mice described in **B** (**P = 0.002, unpaired, 2-tailed *t* test). (**E**) Ingenuity Pathway Analysis of NRPV7^+^ CD8^+^ T cells in remitter versus prediabetic NOD mice (*z* scores ≤–2, *P* < 0.05). (**F**–**H**) Violin plots showing single-cell expression levels of Tcf7, Klf2, and Tox in NRPV7^+^ CD8^+^ T cells (*n* = 4–5 mice/group). *P* values reflect pairwise comparisons. (**I**) Representative flow plots showing population of islet NRPV7^+^TOX^+^ cells among CD8^+^ T cells. (**J**) Summary bar charts showing percentage of TOX expression among NRPV7-tet^+^ CD8^+^ T cells in the islets and ppLNs of anti-CD3–treated versus untreated prediabetic NOD mice. Each symbol represents 1 mouse. All data in summary bar charts are shown as mean ± SEM.

To specifically identify the effects on autoantigen-specific cells, we studied CD8^+^ T cells that recognize NRPV7^+^, residues 206–214 of IGRP, using the TCR Vβ chain sequence as a barcode. We identified likely clonotypes of IGRP-specific CD8^+^ T cells by matching our single-cell TCR sequences with that of published datasets (15). We found a similar frequency of the NRPV7-recognizing CD8^+^ T cells in the islets of remitters and prediabetic NOD mice by scRNA-Seq (Figure 5A) or by flow cytometry (Figure 5B). In both groups of mice, the frequency of IGRP-specific CD8^+^ T cells was higher in the islets than in the ppLNs.

There were 690 DEGs in the IGRP-specific CD8^+^ T cells from the anti-CD3–treated remitters compared with these cells in the islets of prediabetic mice (Figure 5C and Supplemental Table 7). As in the other CD8^+^ T cells, the expression of granzyme B was significantly reduced in the islet NRPV7^+^ cells after treatment (Figure 5D), which we confirmed by flow cytometry. An IPA analysis revealed downregulated pathways associated with mitochondrial activity, antigen presentation, IFN and TNF signaling, apoptosis, and cell cycle (Figure 5E). Neddylation associated with CD8^+^ T cell proliferation, cytokine production, and cytotoxic activity was also significantly reduced (*z* score = –3.9, *P* < 0.0001) (16).

In the prediabetic mice and consistent with previous reports by Gearty et al., the NRPV7-specific cells in the ppLNs had significantly higher expression of *Tcf7* (encoding TCF1) and lower levels of *Entpd1* (encoding CD39) than those in the islets (*P* = 0.0001) (15). However, an increased level of expression of *Tcf7* in these cells in the islets was found after anti-CD3 mAb in comparison with these cells in the islets in prediabetic mice (*P* = 0.0075). The expression levels of *Tcf7* in the antigen-specific cells in the islets were similar to expression in corresponding lymph nodes (Figure 5F). We also observed a similar pattern for transcription factor *Klf2*, which sustains ground-state pluripotency (Figure 5G) (17).

Expression of *Tox* in the islet-infiltrating IGRP-specific CD8^+^ T cells was also significantly increased after anti-CD3 mAb treatment versus prediabetic mice (Figure 5, C and H). We confirmed the higher expression by flow cytometry (Figure 5, I and J). The single-cell analysis showed that the subset of islet-infiltrating IGRP-specific CD8^+^ cells that coexpressed *Tcf7* and *Tox* was significantly higher in remitter mice compared with prediabetic mice, also confirmed by flow cytometry (*P* = 0.04) (Supplemental Figure 6).

### Differentiation of T cells after anti-CD3 mAb treatment.

**Figure 6:**
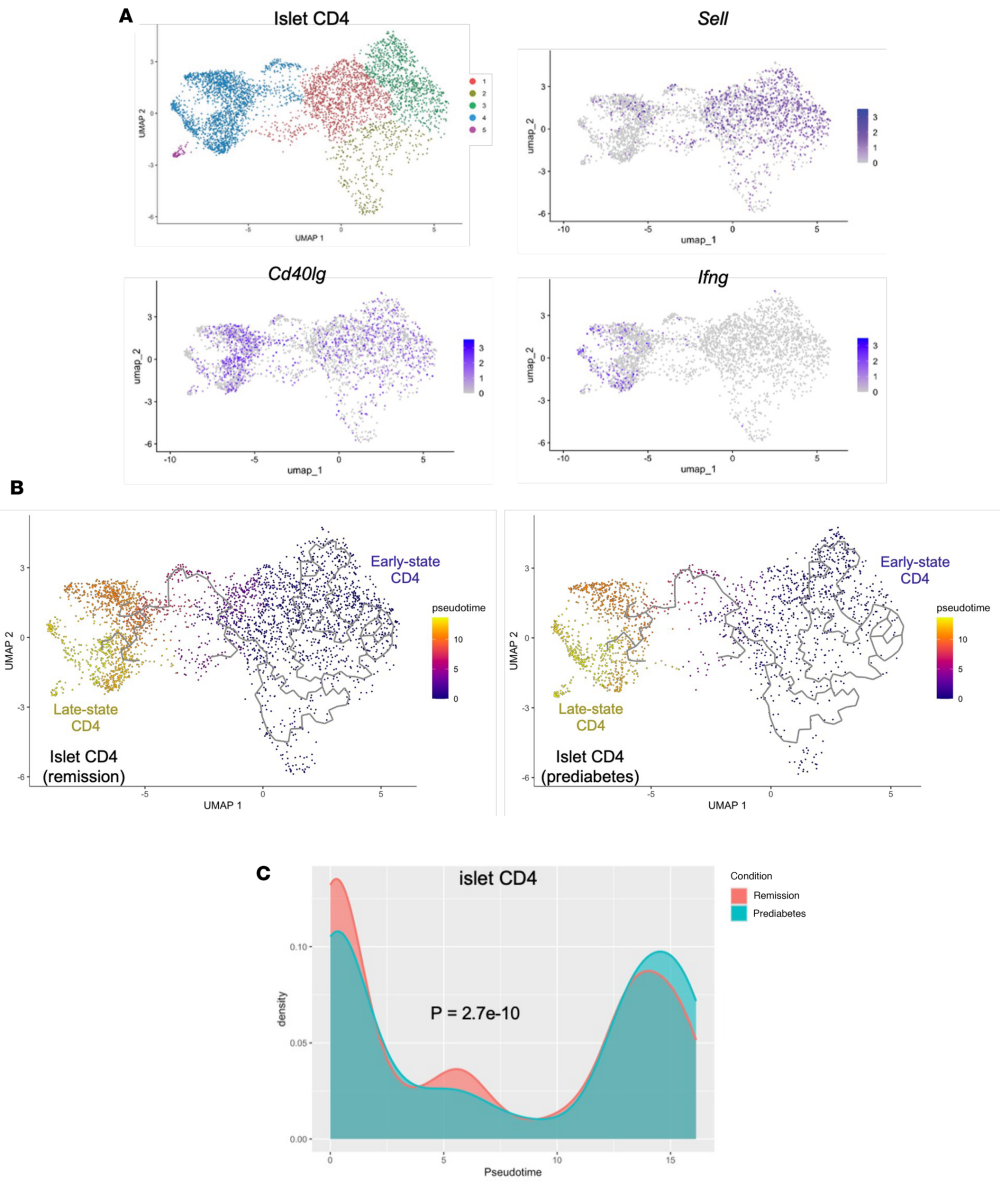
Differentiation of islet CD4^+^ T cells is modified by anti-CD3 treatment. (**A**) UMAP showing clustering of islet CD4^+^ T cells and feature plots showing expressions of *Sell*, *Cd40lg*, and *Ifng*. (**B**) UMAP visualization of single-cell trajectory of CD4^+^ T cells in the islets of anti-CD3–treated remitter or prediabetic NOD mice (colors denote pseudotime values). (**C**) Density distribution graph showing how the concentration of CD4^+^ T cells in the islets distributed along a pseudotime trajectory (colors denote conditions). Density values are scaled to 1 (islet, *P* = 2.7 × 10^–10^, asymptotic 2-sample Kolmogorov-Smirnov test; ppLN, *P* = 0.018; asymptotic 2-sample Kolmogorov-Smirnov test).

These findings suggested that the differentiation of CD4^+^ and CD8^+^ T cells was affected by anti-CD3 mAb treatment. To assess the trajectory of cell differentiation, we performed a pseudotime analysis of the 2 cell subtypes. Islet-infiltrating CD4^+^ T cells were separated into 5 subsets by clustering using Monocle 3 (Figure 6A). Feature plots showed that subsets 1, 2, and 3 were at an earlier stage of differentiation, evident by expression of *Sell* (CD62L) and other genes, whereas subsets 4 and 5 were at later stages and showed signs of activation and effector function as evident by expression of genes such as *Cd40lg* (CD40L) and *Ifng* (IFN-γ) (Figure 6A). The same clustering and expression patterns were used for the paired lymph nodes (Supplemental Figure 4A). We defined pseudotime trajectory to start at early stages 1–3 and continue to late stages 4–5 (Figure 6B). In the islets, there was a statistically significant difference in the distribution of the cells when the remitters were compared with the prediabetic mice using Kolmogorov-Smirnov test (*P* = 2.7 × 10^–10^) (Figure 6C), with a lower cell density at late state in the remitters. The differences in the corresponding ppLNs were also statistically significant (*P* = 0.018) but less than in the islets (Supplemental Figure 4C).

**Figure 7:**
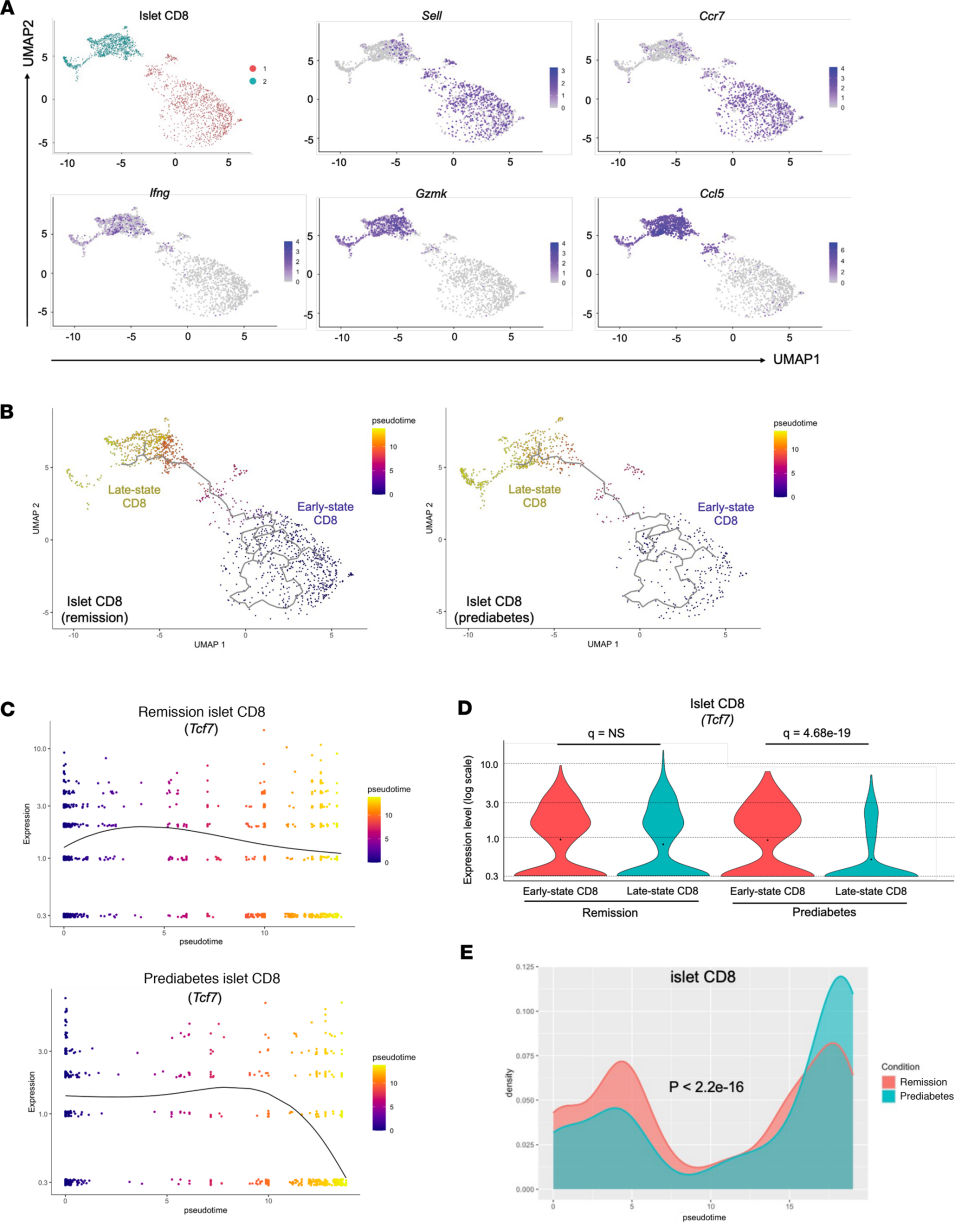
Differentiation of islet CD8^+^ T cells is modified by anti-CD3 treatment. (**A**) UMAP showing clustering of islet CD8^+^ T cells and feature plots showing expressions of *Sell*, *Ccr7*, *Ifng*, *Gzmk*, and *Ccl5*. (**B**) UMAP visualization of single-cell trajectory of CD8^+^ T cells in the islets of anti-CD3–treated remitter or prediabetic NOD mice (colors denote pseudotime values). (**C**) *Tcf7* expression of islet CD8^+^ cells along trajectory in the 2 conditions (expression level shown on a log scale starting from 0.3). (**D**) Violin plots showing single-cell expression of *Tcf7* in early- or late-state islet CD8^+^ T cells from remitter and prediabetic NOD mice (expression level shown on a log scale starting from 0.3; filled dot in each violin plot represents the mean value of expression). (**E**) Density distribution graphs showing how the concentration of CD8^+^ T cells in the islets distributed along a pseudotime trajectory (colors denote conditions). Density values are scaled to 1 (islet, *P* < 2.2 × 10^–16^, asymptotic 2-sample Kolmogorov-Smirnov test).

The CD8^+^ T cells in the islets were separated into 2 subsets by clustering using Monocle 3, in which subset 1 was enriched with expression of genes such as *Sell* (*CD62l*) and *Ccr7*, whereas subset 2 was enriched with effector genes, such as *Ifng*, *Gzmk*, and *Ccl5* (Figure 7A). We defined pseudotime trajectory from subset 1 (early-state) to subset 2 (late-state) (Figure 7B and Supplemental Figure 5B). There was a significant difference in the distribution of CD8^+^ cells in the remitter compared with the prediabetic mice with a greater proportion of cells in the early state versus late state using Kolmogorov-Smirnov test (*P* < 2.2 × 10^–16^) (Figure 7E). In prediabetic mice, *Tcf7* expression declined between the early- and late-state cells (*q* = 4.68 × 10^–19^), whereas it was unchanged in the remitters (Figure 7, C and D). The same clustering and expression patterns were used for the CD8^+^ T cells from ppLNs (Supplemental Figure 5A). We did not find this contrasting pattern of *Tcf7* expression in ppLN CD8^+^ cells between the 2 categories of mice (Supplemental Figure 5C).

### Effects of anti-CD3 mAb treatment on β cells.

**Figure 8:**
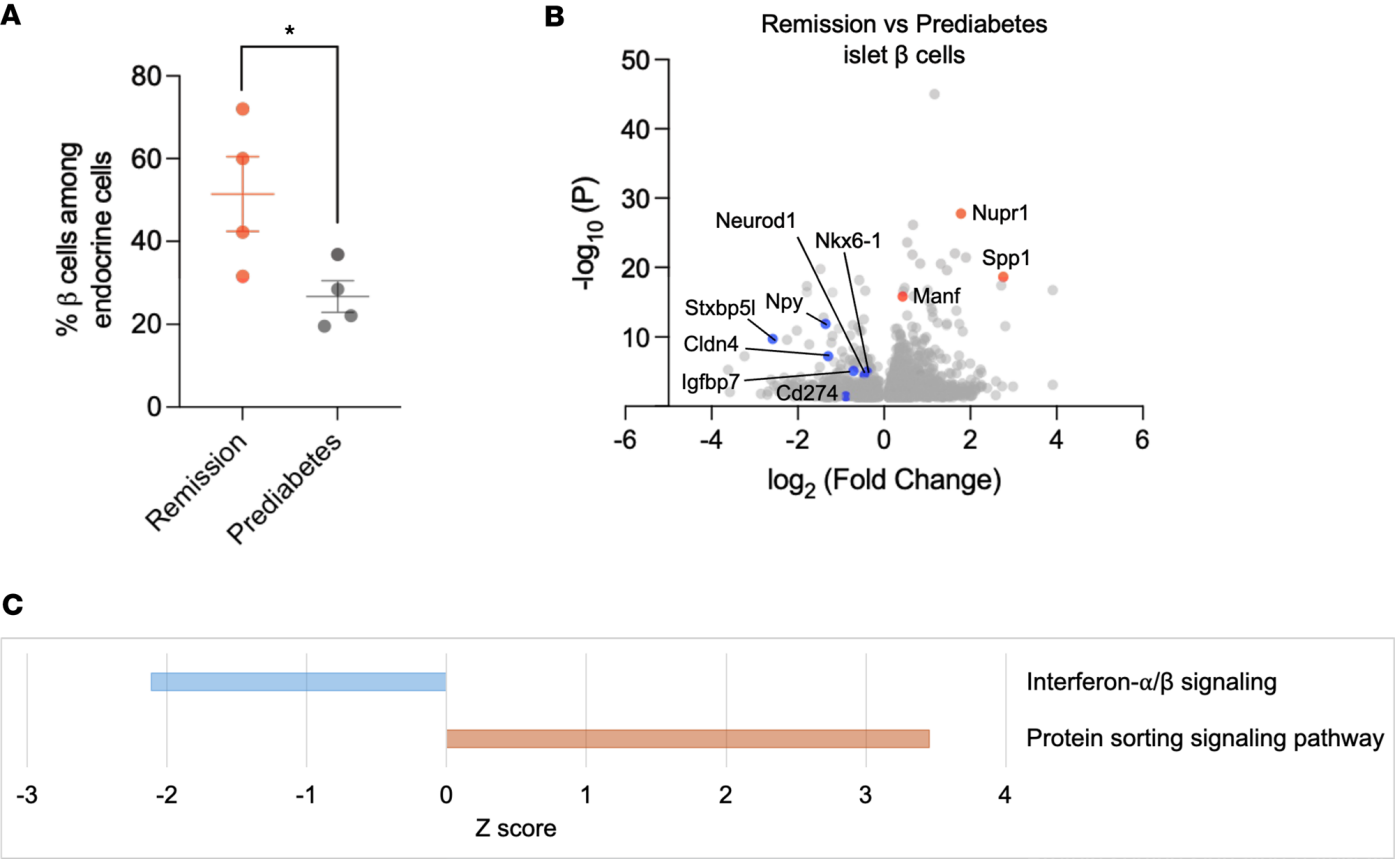
Effects of anti-CD3 mAb treatment on β cells. (**A**) Percentage of β cells among endocrine cells in the islets of remitter or prediabetic NOD mice by scRNA-Seq analysis. Each symbol represents 1 individual mouse (*P = 0.045, unpaired, 2-tailed *t* test). Data are shown as mean ± SEM. (**B**) Volcano plot showing DEGs of β cells in the islets of anti-CD3–treated remitter versus prediabetic NOD mice (*P* values < 0.05). Upregulated genes of note in remitters are highlighted in red and downregulated genes in blue. (**C**) Ingenuity Pathway Analysis of β cells in the islets of remitter versus prediabetic NOD mice (*P* values < 0.05).

The changes in immune cells by anti-CD3 mAb may have affected β cells, which are also a source of inflammatory mediators. The β cells were identified by expression of canonical markers (*Ins1*, *Ins2*). The frequency of β cells among endocrine cells in the islets of remitters was higher than that in prediabetic mice (*P* = 0.045), indicating a significantly increased mass of these cells after anti-CD3 mAb treatment (Figure 8A). We found 2,225 DEGs (*P* < 0.05) when the β cells from the anti-CD3–treated NOD remitters were compared with those from the prediabetic mice (Supplemental Table 8). There was significantly increased expression of genes that promote proliferation and survival in the β cells of remitters, such as *Spp1*, *Manf*, and *Nupr1* (Figure 8B) (18–20). Consistent with this, expression of *Cd274* (encoding PD-L1) was also reduced in remitters (log_2_ fold change in remitters: –0.892, *P* = 0.035), but the difference was not statistically significant after FDR correction. IPA analysis indicated that β cells of remitted mice also showed significantly increased expression of genes associated with protein sorting, while expression of genes associated with type I interferon signaling was significantly reduced (Figure 8C).

### The pathogenicity of diabetogenic immune cells from anti-CD3 mAb–treated remitter mice is restrained.

**Figure 9:**
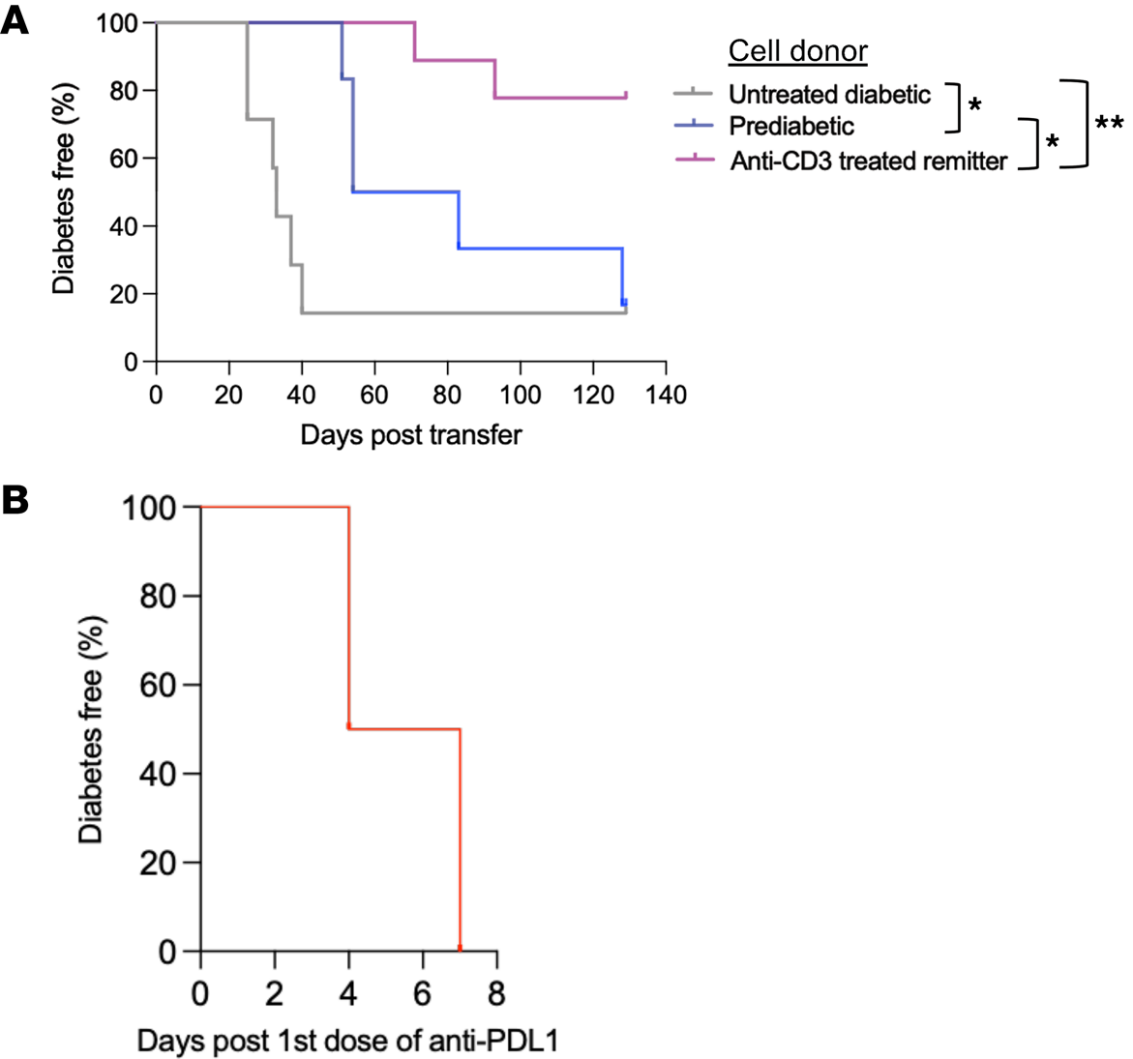
Pathogenicity of diabetogenic immune cells from anti-CD3 mAb–treated remitter NOD mice. (**A**) Spleen cells (15 × 10^6^) from untreated diabetic (gray, *n* = 9), prediabetic (blue, *n* = 6), and anti-CD3–treated remitter (pink, *n* = 7) NOD donors were transferred to NOD/SCID recipients. The time to hyperglycemia is shown. Data are from 3 independent experiments (untreated diabetic vs. prediabetic: *P = 0.045; prediabetic vs. remitter: *P = 0.021; untreated diabetic vs. remitter: **P = 0.001, log-rank tests). (**B**) Anti-CD3–treated remitter NOD mice rapidly developed autoimmune diabetes after treatment with anti–PD-L1 mAb (*n* = 4).

Our studies suggested that potentially diabetogenic T cells persisted in the anti-CD3 mAb–treated mice and had features of T stem cell–like memory (Tscm) cells but were impaired in their ability to cause disease. To test their pathogenic potential, we transferred splenocytes from anti-CD3–treated remitter mice into NOD/SCID recipients. In comparison with splenocytes from untreated diabetic or prediabetic NOD donors, cells from remitters showed a significantly reduced ability to transfer disease into NOD/SCID hosts (remitter NOD vs. prediabetic NOD, *P* = 0.021) (Figure 9A).

We also treated anti-CD3–treated remitter NOD mice with anti–PD-L1 mAb and followed the mice for development of hyperglycemia. We observed a rapid precipitation of autoimmune diabetes in these mice (median time = 5.5 days) (Figure 9B). These results suggest that the diabetogenic T cells are restrained after anti-CD3 mAb treatment but can rapidly precipitate disease if a checkpoint is inhibited.

## Discussion

Our study has identified mechanisms within the islets of Langerhans that lead to operational tolerance to diabetes following anti-CD3 monoclonal antibody (mAb) treatment in NOD mice. We used a treatment regimen of anti-CD3 mAb that modeled the doses used in patients, based on responses and changes in CD8^+^ T cells in the peripheral blood (5, 6). We observed that T cells in the peripheral blood were activated, expressing KLRG1, TIGIT, and PD-1; however, similar to findings in patients, these changes were not long-lasting.

Chatenoud et al. initially reported that T cells, after being cleared from the islets, reappeared within 2 weeks following anti-CD3 treatment (7, 8). Our findings confirm this observation. The T cells that infiltrated the islets after treatment were more heterogeneous with less clonal expansion than was seen in prediabetic mice. The infiltrating cells expressed genes associated with T stem cell–like memory (Tscm) cells, including *Tcf7*, confirmed by increased accessibility in situ using spatial-ATAC-seq (21). These cells exhibited reduced expression of metabolic pathways, suggesting they were not activated by islet antigens but reentered after the depletion of resident effector cells (22).

Despite the more diverse repertoire after anti-CD3 mAb treatment, we found similar frequencies of autoantigen-reactive CD8^+^ T cells in treated and prediabetic mice. Notably, both CD8^+^ and autoantigen-specific CD8^+^ T cells exhibited significantly reduced granzyme B expression, indicative of diminished effector function. CD8^+^ T cells in the islets of the treated mice exhibited higher levels of *Tcf7* and *Klf2* but significantly lower expression of *Entpd1* (CD39), indicating features of Tscm cells. Spleen cells from treated mice could transfer diabetes, albeit at a slower rate than cells from prediabetic or overtly hyperglycemic mice.

Previous studies have shown that autoantigen-reactive CD8^+^ T cells from NOD mice and patients with T1D persist long-term, associated with a stem-like signature (15, 23). TCF1 marks self-renewing T cells that can differentiate into short-lived effector cells capable of β cell destruction in the islets. While the heterogeneous repertoire of islet-infiltrating cells could suggest a mix of naive and memory cells, their ability to transfer diabetes and the quick induction of disease by a checkpoint inhibitor indicate that the cells retained pathogenic potential but were prevented from differentiating into effector cells (24).

There were features of the cells that indicate a mixed phenotype. For example, interferon signaling was significantly reduced in the autoantigen-specific cells, as were neddylation, which regulates T cell function through Erk signaling, and expression of *Mki67*, *Nuf2*, *Kif4*, *Stmn1*, and *Birc5* in all CD8^+^ T cells (Figure 4B); and so was oxidative phosphorylation (16, 25). However, there is crosstalk between microenvironments and immune cells that may be different in the islets and in other locations (26). Thus, the reinfiltrating cells showed a hybrid state of stem-like memory and effector cells that has also been described for human memory CD8^+^ T cells (27). We have further analyzed different subsets of T cells identified by unsupervised clustering (Supplemental Figure 2). However, we did not find significant differences in cell numbers or gene expressions in any T cell subset between the 2 conditions. Therefore, our data suggest that anti-CD3 mAb treatment elicits a global effect on T cells.

Interestingly, the autoantigen-reactive CD8^+^ T cells expressed significantly higher levels of *Tox*, possibly suggesting functional exhaustion, but Tox expression in inflammatory conditions does not necessarily indicate exhaustion. *Tox* expression has been found together with TCF1 on CD4^+^ and CD8^+^ T cells in autoimmune settings and on human effector memory CD8^+^ T cells (28). We did not find significantly higher expression of CD95 (*Fas*) nor CD38 expression after anti-CD3 mAb treatment when the cells in the islets of remitters were compared with those of prediabetic mice. The absence of a detectable difference in CD95 expression may reflect a similarly high level of expression in both settings. Previous studies showed that retention of TCF1 expression promotes persistence and survival of islet-infiltrating CD4^+^ T cells, some of which coexpress *Tox* during the development of autoimmune diabetes in NOD mice (29). These finely tuned cells maintain function and persist during chronic antigen exposure. Further studies will be needed to understand whether the restrain on the function of pathogenic TCF^+^ CD8^+^ T cells is because of the expression of TOX or reflects a heterogeneous population. In addition, the phenotypes of the cells may be affected by their location. For example, we found that the expression of *Tcf7* was increased 1.45-fold in CD8^+^ T cells from the peripheral blood from teplizumab- versus placebo-treated participants at 3 months in the teplizumab prevention trial, but *Tox* was not (4).

By necessity our studies are cross-sectional, and therefore we cannot track the fate of individual cells before, during, and after treatment. Most likely, T cell activation was required for the changes, since the FcR non-binding mAb teplizumab was found to cause T cell activation, and treatment with cyclosporin A can prevent the effects of the anti-CD3 mAb (7). In addition, we found signs of cellular activation among CD4^+^ T cells in the ppLNs. The signal that is delivered, however, is partial, which prevents full development of effector cells. Our data with a checkpoint inhibitor indicate that, depending on the balance of agonist and regulatory signaling, the cells can be rapidly induced to become effectors or Tscm-like.

Our most striking findings were in CD8^+^ T cells, but there were similar changes among CD4^+^ T cells to a lesser degree. This is not surprising since our own studies have shown a much greater effect of FcR non-binding anti-CD3 mAb on tolerization of CD8^+^ than CD4^+^ T cells (3). We did not find significant changes in conventional CD4^+^ Tregs in the islets or in the draining lymph nodes. Previous investigations have described sparing of Tregs with anti-CD3 mAb, and others have identified TGF-β–producing Tregs in the draining lymph nodes of treated mice (22). In addition, in those studies the CD4^+^CD25^+^ Tregs from anti-CD3 mAb–treated mice protected them from diabetes transfer (30). Further adoptive transfer studies would be needed to confirm these properties.

Our findings also indicated differences in β cells between remitter and prediabetic mice, with remitter mice showing increased cell frequency (Figure 8A) and reduced interferon signaling (Figure 8C). Curiously, like our findings in T cells, the expression of immune-inhibitory molecules (e.g., PD-L1) was reduced. Most likely this is the consequence of the reduced type I interferon signaling.

There are limitations to our studies. Unlike patients with and at risk for T1D, who are genetically and immunologically heterogeneous, our studies were in an inbred mouse strain. Therefore, caution is needed in generalizing our findings to the effects of treatment in patients. Second, there is an age difference in the groups. A comparison with age-matched mice that had not developed hyperglycemia would not be appropriate since, in older non-diabetic mice, the mechanisms that led to the development of hyperglycemia did not occur. Likewise, there were not recoverable islets from age-matched mice that had developed hyperglycemia at the same age as the mice with anti-CD3 mAb–induced remission. In addition, we did not find a significant difference in the CD8^+^ T cell entropy between the islets from younger (15–17 weeks) prediabetic mice and older (30–35 weeks) NOD mice without diabetes. Thus, our analysis essentially is a comparison of islet-infiltrating cells before and after anti-CD3 mAb. Moreover, our TCR entropy score was higher in the anti-CD3 mAb–treated mice than in the younger prediabetic mice, which would not have been predicted in older mice.

We analyzed autoreactive T cells of a single specificity, and the effects on other autoreactive cells may differ. In addition, we cannot accurately quantitate the entire autoreactive T cell pool and, because of technical limitations, were unable to directly assess the autoreactivity of the cells that infiltrate the islets after anti-CD3 mAb. T cells that are found in the islets of remitter NOD mice might be skewed to low avidity, or there may be other selection factors. Nonetheless, our findings in autoreactive T cells in the peripheral blood from patients suggest that the autoantigen-reactive T cells are prevented from proliferation with teplizumab treatment, and are consistent with our previous studies (4).

In summary, we have identified changes that occur in the islets after anti-CD3 mAb treatment that may explain the prolonged efficacy that may be seen in humans. The treatment reshapes the islet-infiltrating repertoire, resulting in islet-infiltrating cells with a more diverse repertoire, with some features of Tscm cells but other features that indicate a hybrid phenotype including characteristics of effector T cells. This finding reconciles with our results that autoreactive T cells persist in a tenuous state after anti-CD3 treatment and can be rapidly activated to cause disease by disturbing the balance with a checkpoint inhibitor. Finally, our studies have identified discrepancies between the peripheral blood and the tissue and suggest that the early features in T cells after anti-CD3 mAb treatment in the blood may not account for the long-term clinical responses. Further detailed studies to relate findings in the peripheral blood to those in the islets may help to guide future therapies with anti-CD3 mAb.

## Methods

### Sex as a biological variable.

All the mice in this study were female because female NOD mice spontaneously develop autoimmune diabetes at a significantly higher incidence and with earlier onset than male mice. These findings are expected to be relevant for both male and female mice with diabetes.

### Mice.

Female NOD/ShiLtJ (WT NOD) and NOD.Cg-*Prkdc*^scid^/J (NOD/SCID) mice were purchased from The Jackson Laboratory. The mice were maintained in the animal facility under specific pathogen–free conditions at Yale University.

### Anti-CD3 mAb treatment.

Glucose levels were measured twice weekly beginning at 10 weeks of age. NOD mice with blood glucose measurements higher than 250 mg/dL for 2 consecutive days were considered diabetic and treated with F(ab′)_2_ fragments of anti-CD3 mAb 145-2C11 (5 μg/d administered i.p.) for 3 or 4 consecutive days. Random glucose levels were measured in the peripheral blood by handheld meter. Diabetic NOD mice after anti-CD3 mAb treatment with normoglycemia for ≥9 weeks were considered in long remission.

### Flow cytometric analysis.

Peripheral blood mononuclear cells were isolated using eBioscience Red Blood Cell Lysis Buffer (Invitrogen). Pancreatic draining lymph nodes and islets were processed into single cells following previously published protocols (15, 31). They were counted using a hemocytometer and stained with LIVE/DEAD Fixable Yellow Dead Cell Stain (Invitrogen). Cells were then incubated with anti–mouse CD16/32 antibody (clone 93, BioLegend) for Fc receptor blocking and stained with surface antibodies PerCP–anti–mouse CD45 (clone 30-F11, BioLegend), APC-eFluor780–anti–mouse CD3 (clone 17A2, eBioscience), Brilliant Violet 711–anti–mouse CD4 (clone GK1.5, BioLegend), Alexa Fluor 700–anti–mouse CD8a (clone 53-6.7, BioLegend), Brilliant Violet 421–anti–mouse PD-1 (clone 29F.1A12, BioLegend), PE–anti–mouse/human KLRG1 (clone 2F1/KLRG1, BioLegend), and PE/Cyanine7–anti–mouse TIGIT (clone 1G9, BioLegend). IGRP-specific CD8^+^ T cells were identified using MHC class I tetramer APC H-2K^d^/NRPV7 (KYNKANVFL) purchased from MBL. Intracellular transcription factor staining was performed using the FOXP3 Fix/Perm Buffer Set (BioLegend) with Alexa Fluor 488–anti-TCF1 (C63D9, Cell Signaling Technology) and PE–anti–human/mouse TOX (clone REA473, Miltenyi Biotec) per the manufacturer’s protocols. Flow cytometric data were acquired using LSRFortessa (BD Biosciences) and analyzed with FlowJo v10 software (BD Biosciences).

### Assessment of insulitis.

Pancreata isolated from NOD mice were embedded in Tissue-Tek OCT Compound (Sakura Finetek USA Inc.), frozen in cryomolds, and cryosectioned at 3 tissue depths. Sectioned slices were stained with hematoxylin and eosin (H&E) and assessed for insulitis based on a 4-grade scoring system: 0, no insulitis (normal islet); 1, peri-insulitis (<10% of the islet infiltrated); 2, 10%–50% of the islet infiltrated; 3, >50% of the islet infiltrated.

### Adoptive cell transfer.

Six-week-old female NOD/SCID mice were used as recipients. Spleen cells from diabetic or prediabetic NOD mice without treatment or those showing stable remission after treatment with F(ab′)_2_ fragment of anti-CD3 mAb were collected and processed into single cells and frozen. The cells were thawed, and 15 × 10^6^ splenocytes were administered via i.p. injection to NOD/SCID recipients. Mice were then monitored and considered diabetic with blood glucose measurements of greater than 250 mg/dL for 2 consecutive days.

### Anti–PD-L1 treatment.

Anti-CD3 mAb–treated remitter NOD mice were treated with 100 μg anti–PD-L1 (clone 10F.9G2, Bio X Cell, catalog BP0101 or BE0101) every 4 days for 2 doses. Mice were monitored daily for hyperglycemia.

### Single-cell transcriptome sequencing.

Pancreatic lymph nodes and islets were isolated from each of the NOD mice and dispersed into single cells. After washing and counting, each cell sample was incubated with one of the anti-mouse Hashtag antibodies (TotalSeq-C0301-3, BioLegend, catalog 155861, 155863, 155865) to enable multiplexing. Hashtagged cell samples were pooled for 10x Genomics single-cell sequencing conducted by the Yale Center for Genome Analysis. Single-cell VDJ and 5′ gene expression libraries were generated using Chromium Next GEM Single Cell 5′ Reagent Kits v2 (Dual Index) with Feature Barcode technology. Post-library-construction QC was assessed using Agilent TapeStation traces. Library sequencing was done using a Hiseq run of 100 bp paired-end reads by NovaSeq (Illumina). In the analysis of islet-infiltrating cells, immune cell clusters were confirmed by expression of *Ptprc* (CD45).

### Single-cell RNA and single-cell TCR analyses.

Raw sequencing reads were processed using the CellRanger pipeline (v7.1.0). Single-cell 5′ expression reads were aligned to reference transcriptome mm10-2020-A. All transcriptomic analysis was built under R version 4.3 using the Seurat package (v5.0.0). Low-quality cells (with <200 genes and >5% mitochondrial genes) and empty droplets were filtered out. Demultiplexing was performed by hashtagged oligonucleotides to distinguish individual samples, and data normalization was done using the CLR method. Ambient RNA contamination in islet cell samples was removed and corrected by SoupX (https://cran.r-project.org/web/packages/SoupX/). Then the scDblFinder package was used to select singlets from the dataset for downstream analysis. After pre-processing of each individual sample, all datasets were merged into one Seurat object, and then Harmony Integration was run. Dimensionality reduction was performed using Harmony embeddings in Seurat. Cell clusters were visualized using uniform manifold approximation and projection (UMAP). Differential gene expression analysis was done with DEG analysis in Seurat. For comparisons of CD4^+^ and CD8^+^ T cells, adjusted *P* values were used. Because of low cell numbers, for the analysis of IGRP-specific CD8^+^ T cells and β cells, raw *P* values were used. TCR analysis was conducted using the scRepertoire package. TCR clones were counted, and Shannon entropy was calculated using the posterior package. Single-cell trajectory analysis was performed using the Monocle 3 package (https://cole-trapnell-lab.github.io/monocle3/).

### Spatial-ATAC-seq analysis.

Pancreas tissues isolated from anti-CD3 mAb–treated remitter NOD or prediabetic NOD mice were embedded in Tissue-Tek OCT Compound (Sakura Finetek USA Inc.) and frozen in cryomolds. The frozen tissue block was sectioned into approximately 7 μm thickness and placed in the center of a poly-l-lysine slide (Electron Microscopy Sciences, catalog 63478-AS), then immediately stored at –80°C as previously described (32). Spatial-ATAC-seq was performed following a standard protocol (33). Briefly, the tissue was first fixed with 0.2% formaldehyde and permeabilized. Tagmentation was carried out using Tn5 transposase, followed by 2 rounds of barcode ligation. The barcoded DNA fragments were then collected, and a sequencing library was constructed via PCR. The library was sequenced on an Illumina NovaSeq X with a paired-end 150 bp setup. A gene score matrix was generated using the software package ArchR for chromatin accessibility analysis (34).

### Statistics.

All data are presented as mean ± SEM unless otherwise indicated. Comparisons between groups were done by 2-tailed *t* test, χ^2^ test, or Mann-Whitney test using GraphPad Prism version 10. Corrections were made for multiple comparisons as indicated. *P* values of less than 0.05 were considered significant.

### Study approval.

All protocols were approved by the Yale Institutional Animal Care and Use Committee.

### Data availability.

Values for all data points in graphs are reported in the Supporting Data Values file. Single-cell RNA-Seq raw data were deposited in the NCBI’s Gene Expression Omnibus under accession number GSE292313.

## Author contributions

YW designed and conducted studies, analyzed all the data, and wrote the manuscript. MS and ALD analyzed scRNA-Seq data and wrote the manuscript. SD and MNB performed animal studies. DW and YL performed spatial-ATAC-seq studies and analyzed data. ALP analyzed data and designed studies. KCH designed studies, analyzed data, and wrote the manuscript.

## Funding support

This work is the result of NIH funding, in whole or in part, and is subject to the NIH Public Access Policy. Through acceptance of this federal funding, the NIH has been given a right to make the work publicly available in PubMed Central.

- NIH R01-DK057846 to KCH.
- Breakthrough T1D 2-SRA-2022-1197-S-B to KCH.

## Supplementary Material

Supplemental data

Supplemental tables 1-8

Supporting data values

## Version 1 01/23/2026

Electronic publication
