# Supplementary material for: Anti-CD3 mAb treatment reshapes infiltrating T and β cells in the islets in autoimmune diabetes
Source: JCI Insight. 2026 Jan 23;11(2):e192755. doi: 10.1172/jci.insight.192755 (PMC12892913; doi:10.1172/jci.insight.192755)
Supplement: Supplemental data [file jciinsight-11-192755-s210.pdf]

Supplemental Figure 1

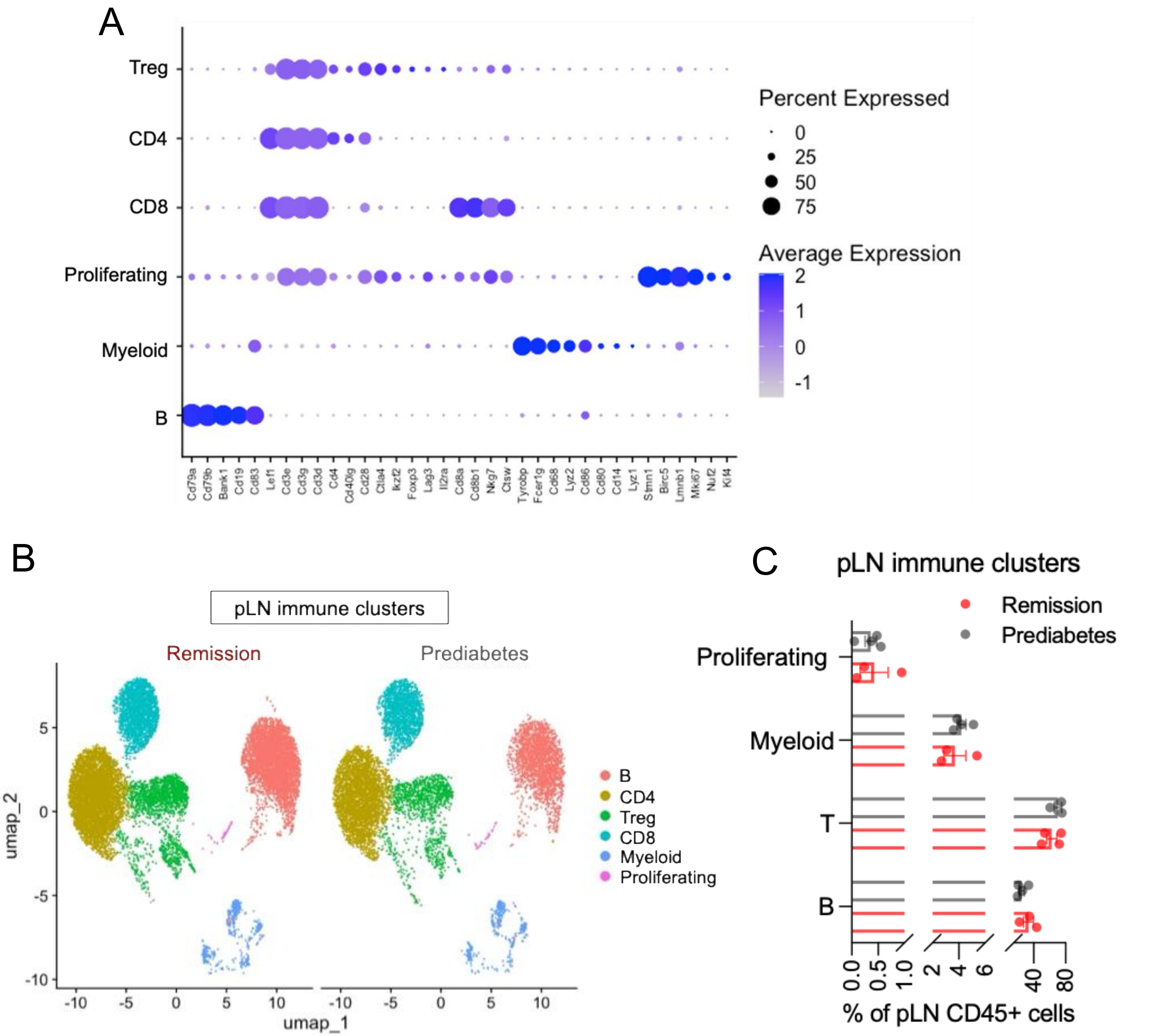

Figure S1. (A) Dot plot showing single-cell expression of canonical markers used to annotate immune clusters. (B) UMAP showing immune cell clusters in the pancreatic lymph nodes (pLN). (C) Percentage of each immune cluster among pLN CD45+ cells.

Supplemental Figure 2

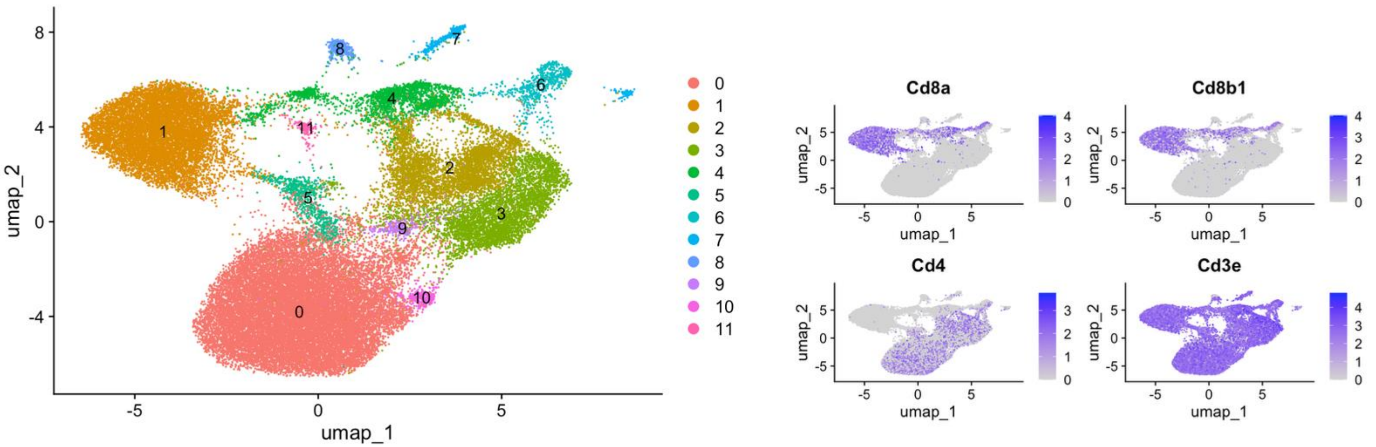

0: Naïve/memory Cd4
1: Naïve/memory Cd8
2: Effector Cd4
3: Treg
4: Effector Cd8
5: Egr3+ Cd4
6: Proliferating
7: Gd T cell
8: NK
9: Malat1+ Cd4
10: Interferon stimulated Cd4
11: Other T cells

Figure S2. UMAP showing T cell subsets using unsupervised clustering.

Supplemental Figure 3

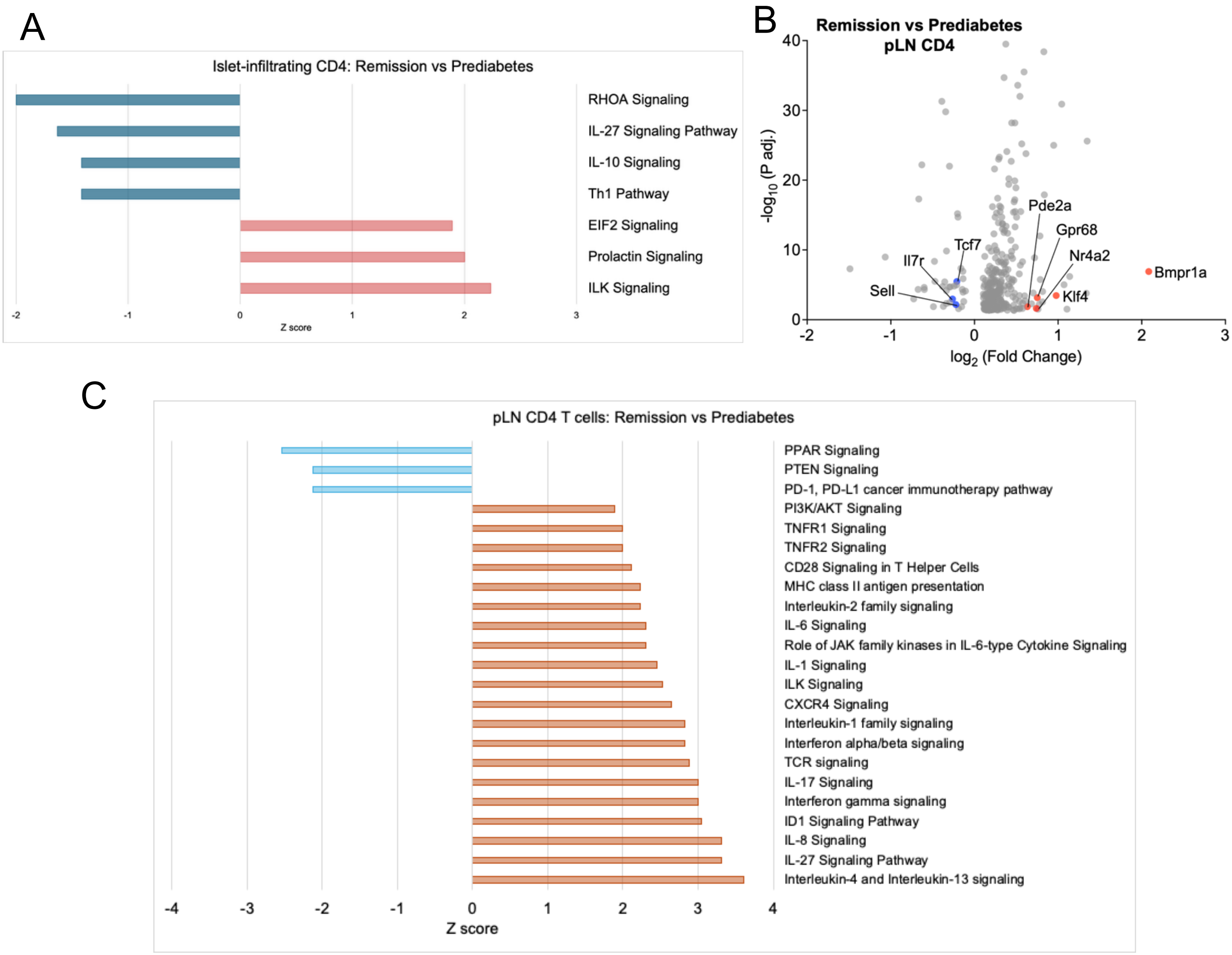

Figure S3. (A) Ingenuity pathway analysis of CD4+ T cells in the islets of remitter versus prediabetic NOD mice (*P* values < 0.05). (B) Volcano plot showing DEGs of CD4 T cells in the pLN of remitter versus prediabetic NOD mice (adjusted *P* values < 0.05). (C) Ingenuity pathway analysis of CD4+ T cells in the pLN of remitter versus prediabetic NOD mice (*P* values < 0.05).

Supplemental Figure 4

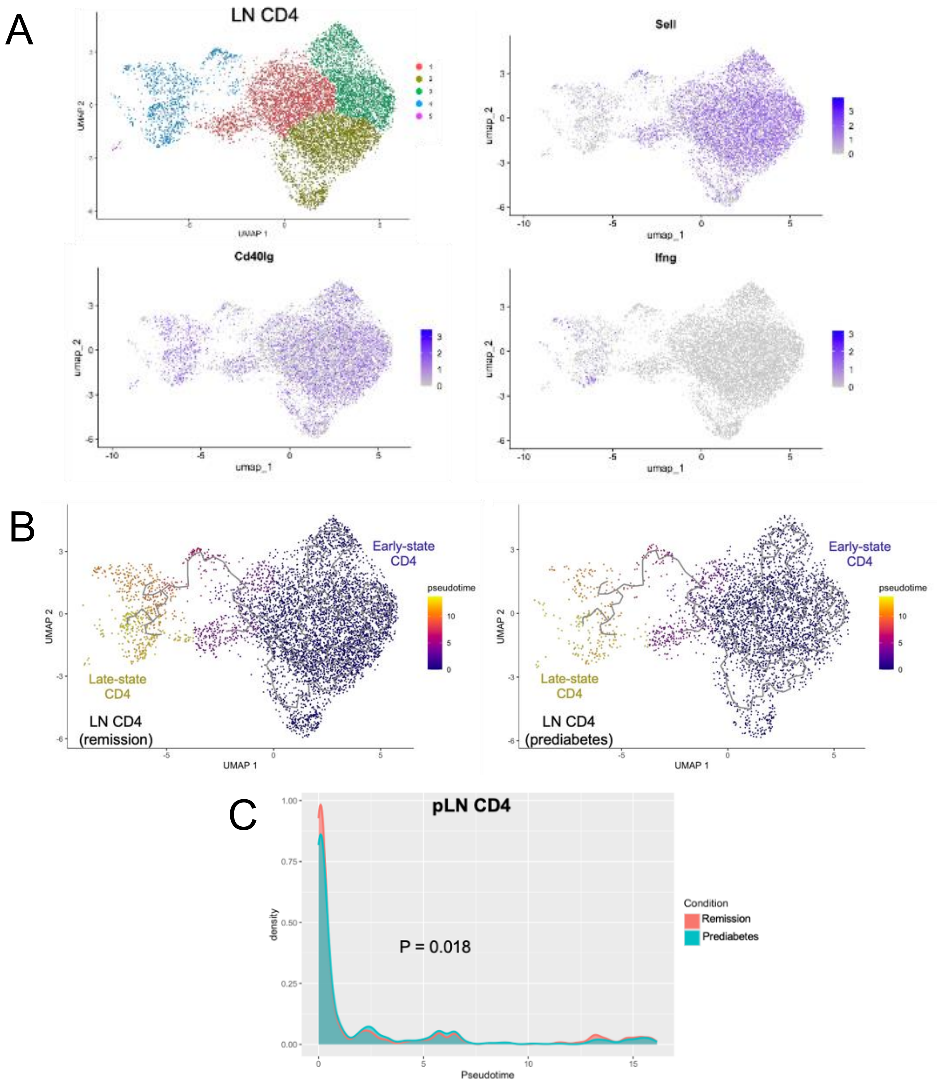

Figure S4. (A) UMAP showing clustering of LN CD4 cells and feature plots showing expressions of Sell, Cd40lg, and Ifng. (B) UMAP showing pseudotime trajectory of LN CD4 cells in the 2 conditions. (C) Density distribution graph showing how the concentration of CD4+ T cells in the LN distributed along a pseudotime trajectory (colors denote conditions). Density values are scaled to 1 (P = 0.018, by asymptotic two-sample Kolmogorov-Smirnov test).

Supplemental Figure 5

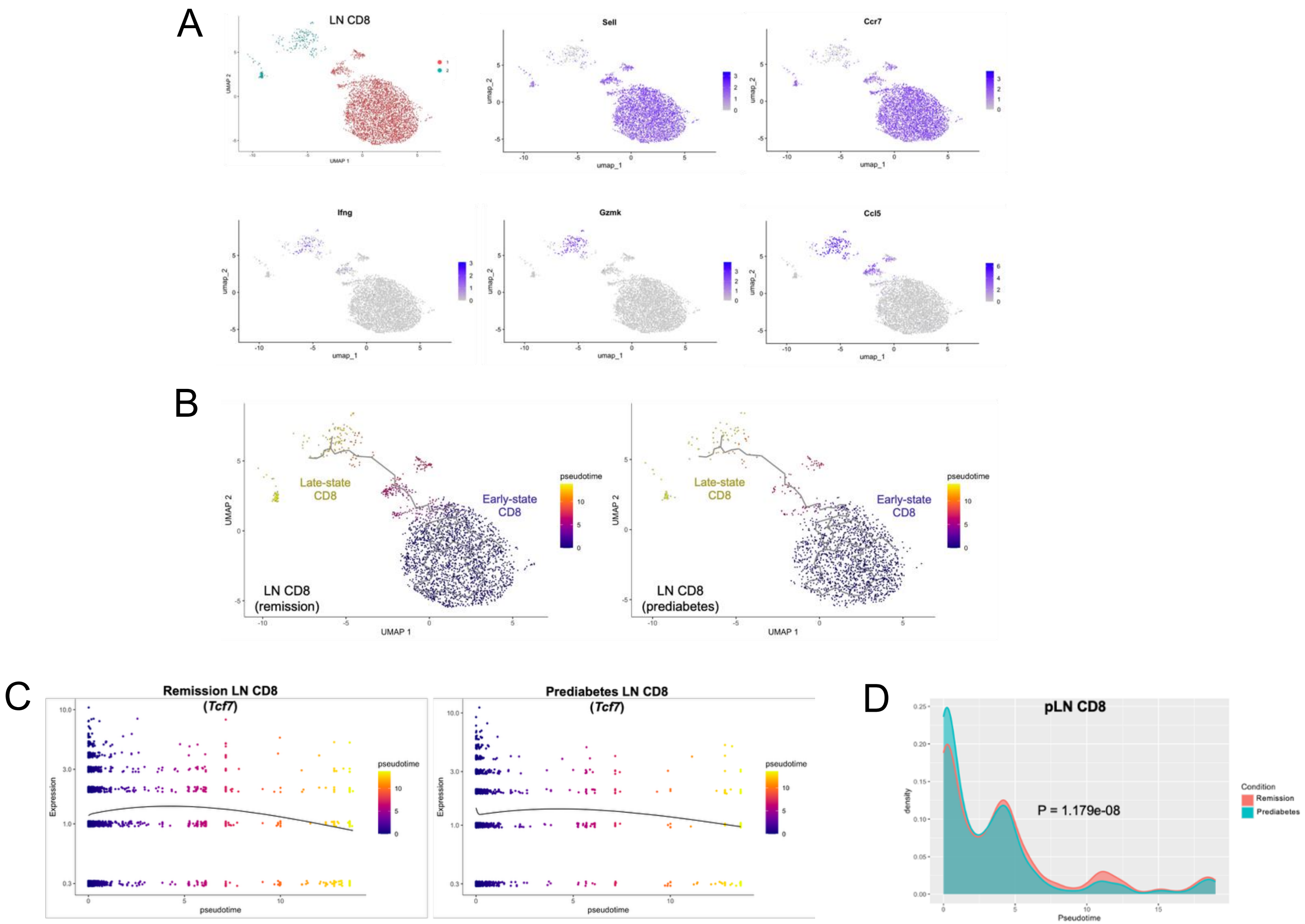

Figure S5. (A) UMAP showing clustering of LN CD8 cells and feature plots showing expressions of *Sell*, *Ccf7*, *Ifng*, *Gzmk*, and *Ccl5*. (B) UMAP showing pseudotime trajectory of LN CD8 cells in the 2 conditions. (C) *Tcf7* expression of LN CD8 cells along trajectory in the 2 conditions. (D) Density distribution graph showing how the concentration of CD8+ T cells in the LN distributed along a pseudotime trajectory (colors denote conditions). Density values are scaled to 1 (P = 1.179e-08, by asymptotic two-sample Kolmogorov-Smirnov test).

Supplemental Figure 6

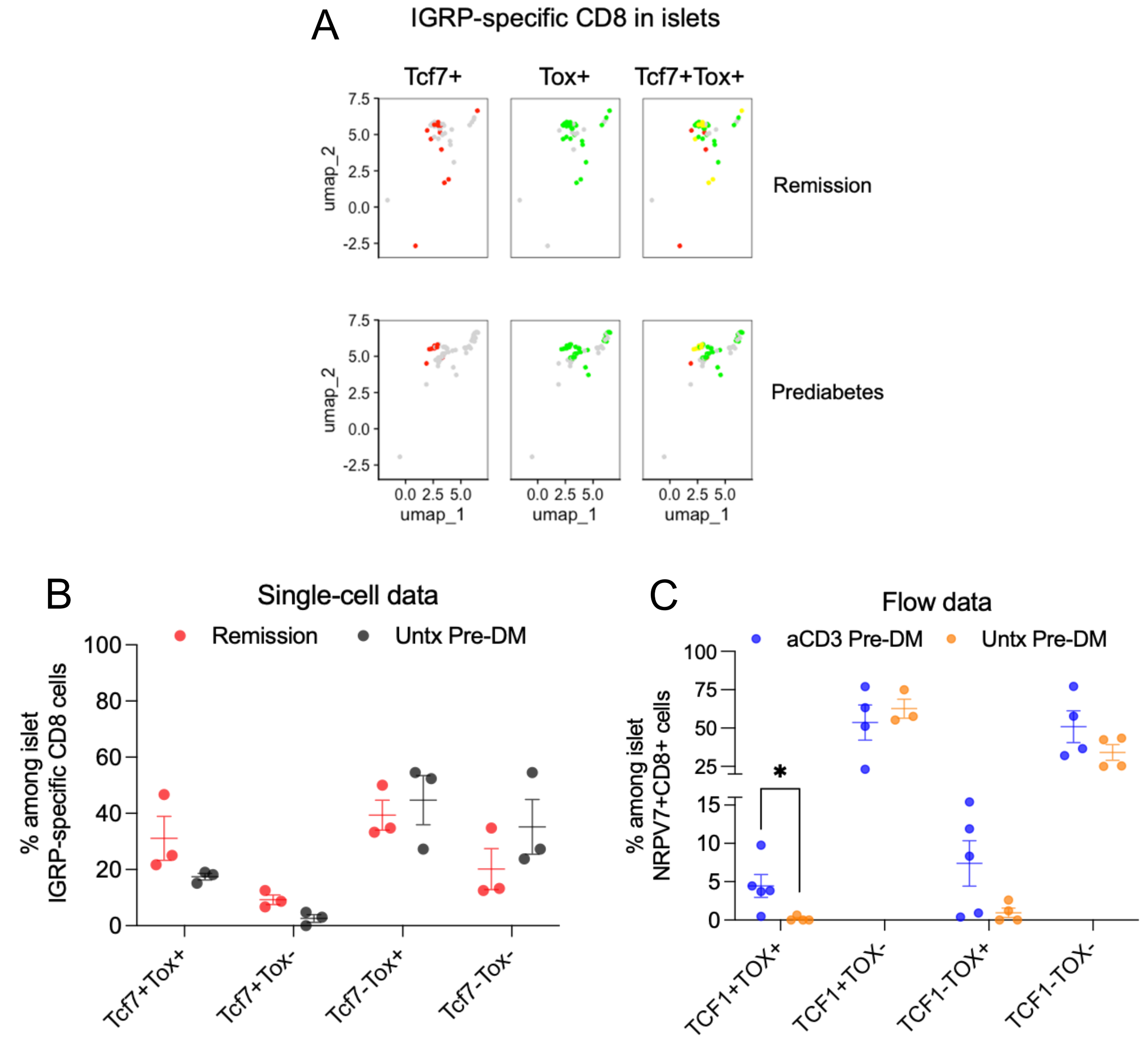

Figure S6. (A) UMAP showing single-cell expression of Tcf7 and Tox in islet IGRP-specific CD8 cells. One dot represents one cell. Red color denotes Tcf7+, green color denotes Tox+, and yellow color denotes Tcf7+Tox+. (B) Single-cell data showing percentage of Tcf7+/- and Tox+/- cells among IGRP-specific CD8 cells in the islets of remitter or prediabetic NOD mice. (C) Flow cytometric analysis showing percentage of TCF1+/- and TOX+/- cells among NRPV7+CD8+ cells in the islets of anti-CD3 treated vs untreated prediabetic NOD mice (*P = 0.04 by unpaired t test).
